# Cooperative Interaction of Phenolic Acids and Flavonoids Contained in Activated Charcoal with Herb Extracts, Involving Cholesterol, Bile Acid, and FXR/PXR Activation in Broilers Fed with Mycotoxin-Containing Diets

**DOI:** 10.3390/antiox11112200

**Published:** 2022-11-07

**Authors:** Ying Zhang, Zishen Lin, Lixue Wang, Xiangyue Guo, Zhihui Hao, Zhen Li, Lee J. Johnston, Bing Dong

**Affiliations:** 1 State Key Laboratory of Animal Nutrition, China Agricultural University, Beijing 100193, China; 2 Center of Research and Innovation of Chinese Traditional Veterinary Medicine, China Agricultural University, Beijing 100193, China; 3 State Key Laboratory of Plant Physiology and Biochemistry, College of Biological Sciences, China Agricultural University, Beijing 100193, China; 4 Swine Nutrition and Production, West Central Research and Outreach Center, University of Minnesota, Morris, MN 56267, USA

**Keywords:** activated charcoal, mycotoxin, meat quality, antioxidants, detoxification

## Abstract

The charcoal–herb extract complex (CHC) is a product of activated charcoal sorption of herb extracts that contain phenolic acids and flavonoids. The effective dose of CHC to promote animal growth is about one tenth of effective dosage of activated charcoal. The purpose of this study was to evaluate potential cooperative interactions between activated charcoal and herb extracts. Two feeding experiments were conducted. In Experiment 1, a responsive dose of CHC to broiler growth was determined to be 250 mg/kg of the diet. In Experiment 2, CHC increased growth performance and improved meat quality, but decreased indices of oxidative stress and inflammation as compared with similar doses of activated charcoal or herb extracts. CHC also increased concentrations of serum cholesterol, bile acid in the gallbladder, and bile acid in feces. The herb extracts present in CHC were largely represented by phenolic acids (PAs, caffeic acid, and vanillin) and flavonoids (FVs, daidzein, and quercetin-D-glucoside) in the detoxification activity of CHC in a mouse rescue test when the mice were gavaged with T-2 mycotoxin. PAs and FVs significantly increased the expression of *CYP7A1*, *PXR*, *CYP3A37*, *Slco1B3*, and *Bsep* in chicken primary hepatocytes. In conclusion, CHC integrated the cooperative interactions of activated charcoal and herb extracts via the FXR/RXR-PXR pathway to detoxify mycotoxins.

## 1. Introduction

The contamination of animal feed by mycotoxins is extensively present in the world, especially in developing countries. About 25% of the world’s feed supply is contaminated with mycotoxins [1]. Supplementation with absorbents such as activated charcoal in broiler feed is an approach to sequester mycotoxins and ameliorate oxidative stress caused by mycotoxins. Commercial broilers endure several conditions that can cause oxidative stress. Fast growth and the associated high metabolic rates cause local hypoxia within the muscle, which leads to the release of reactive oxygen species (ROS) by the mitochondrial electron transport chain. This release of ROS causes oxidative stress. Mycotoxins, metabolites of mycotoxins, and pathogens commonly present in feed can cause the excessive production of free radicals and inflammation in broilers [2,3,4]. Common approaches to mycotoxin mitigation focus on the absorption and degradation of mycotoxins in feed [5]. An alternative approach to reducing the negative effects of mycotoxins might be to increase detoxification and promote the excretion of mycotoxins by the animal.

Activated charcoal can absorb mycotoxins in vitro [6,7], reverse mycotoxin-induced immune suppression [8,9], and improve the growth performance of broilers [10]. Effective doses of activated charcoal range from 0.3% to 10% according to a recent review of studies published between 1980 and 2019 [11]. Dietary supplementation with high levels of charcoal (over 10%) seldom induces negative effects in animals, but some researchers have reported lower feed conversion rates [12,13]. In vitro, charcoal absorbs vitamins and minerals [14,15]. Charcoal or activated charcoal is inert and cannot be digested or absorbed by animals. Coupling the absorptive characteristic of charcoal with a component that is active against mycotoxins could improve the mycotoxin mitigation potential of both components. Many Chinese herbs have antioxidant, anti-inflammatory, and antibacterial properties [16]. Herbs and their extracts have been used widely as feed additives since 2020, when China banned the use of antibiotics in animal feed [17].

The charcoal–herb extract complex (CHC) is a product resulting from the sorption of herb extracts by activated charcoal [18]. CHC has dual functions provided by activated charcoal, which acts as an absorbent, and by Chinese herbs extracts, which provide antibacterial properties. Some Chinese herbs such as *Pulsatilla chinensis*, *Portulaca oleracea* L., *Artemisia argyi Folium*, and *Pteris multifida Poir* contain numerous active components that possess antioxidant and anti-inflammatory activities. These components include: lupine glycosides (from *Pulsatilla chinensis*) [19,20]; alkaloids, oleralignan, syringaresinol, lirioresinol, and monomethyl tetrahydroxy truxinate (from *Portulaca oleracea* L.) [21,22]; artemilinin, sesquiterpene-monoterpene lactone, and isoartemisolide (from *Artemisia argyi Folium*) [23]; and pterosin dimers and flavoids (from *Pteris multifida Poir*) [24,25].

Coupling active compounds of Chinese herbs with activated charcoal can confer antioxidant and anti-inflammation activities on CHC. In piglets, the effective dosage of CHC for growth promotion ranged from 0.05% to 0.1% [18]. In our previous study of chickens, the effective CHC doses were from 0.05% to 0.075% [26]. These effective doses of CHC are about one-tenth of effective dosage of activated charcoal that has been shown to improve the performance of birds [12,13,27,28,29,30] or pigs [31,32,33,34,35]. These meaningful differences in doses of activated charcoal and CHC suggest the cooperative interactions between activated charcoal and herb extracts.

The study reported herein was designed to characterize CHC’s functions in vitro and investigate its effects on growth performance, immune status, and the meat quality of broilers in vivo. The mechanism of the cooperative interactions of activated charcoal and herb extracts was revealed by comparing the effect of CHC on activated charcoal and herb extracts separately. The bioactive components of the herb extracts were quantified, and the identified compounds representing the functions of the herb extracts were investigated.

## 2. Materials and Methods

### 2.1. Preparation of Charcoal and Herb Extracts

The manufacture of CHC followed the procedures of Wang et al. [18], with modifications. In brief, CHC was produced by the active charcoal sorption of Chinese herb extracts (CHC-Herb). Activated carbon was prepared by crushing and screening cedar wood and pine wood, followed by carbonization and activation at high temperatures (500~900 °C). The activated carbon was screened, and particles ranging from 0.18 to 0.25 mm in size were retained. CHC had a surface area of 2800 m^2^/g. The mixture of traditional Chinese herbs included raw plants of *Pulsatilla chinensis* (dried rhizome), *Portulaca oleracea* L. (dried whole plant), *Artemisia argyi Folium* (dried whole plant), and *Pteris multifida Poir* (dried whole plant) at the ratio of 7:8:10:6. The plant mixture (1 kg) was ground and mixed in 60% ethanol (1 L). Subsequently, the volume was increased five-fold with the use of water, and the mixture was incubated for 12 h. The plant mixture was then decocted twice (1 h each) at 100 °C under the standard atmosphere pressure. After being filtered, the decoction was collected and concentrated to about 0.2-fold volume of the initial plant volume of CHC-Herb at 55 °C under low pressure. The proportion of herb extracts (drug extract ratio) was 1000 g:1000 mL. Following thorough drying at 55 °C under low pressure, the herb extracts were weighed out, and the extraction rate was calculated to be at 11%, which meant that 1000 g raw herbs yielded 110 g dry extracts. Concentration of CHC-Herb was ~1 g/mL. Finally, CHC was produced by mixing CHC-Herb and activated carbon at a proportion of 1 mL (herb extracts): 9 g (activated charcoal) for 8 h, which was then dried under low pressure and ground through 60–mesh screen.

### 2.2. Analysis of CHC-Herb

CHC-Herb was analyzed with a UPLC-HRMS system (UPLC, ACQUITY UPLC H-Class Bio, Waters, Milford, MA, USA); MS, Q-Exactive (Thermo Fisher Scientific, San Jose, CA, USA) equipped with a hearted electrospray ionization (HESI) source (Milford, MA, USA), using the UPLC/MS method as reported by Cao et al. [36]. UPLC separation was performed on a BEH C18 column (2.1 × 100 mm, 1.7 μm, Waters, Milford, MA, USA) at a flow rate of 0.3 mL min^−1^. HPLC grade solvents and additives were from Fisher Scientific (Thermo Fisher Scientific, Bridgewater, NJ, USA). The gradient program using 0.1% FA in water (phase A) and 0.1% FA in ACN (phase B) was applied as follows: 95% A at 0 min up to 55% A at 7 min, 5% A at 10 min and held for 4 min, and then returned to initial condition. The column temperature was 35 °C, and the injection volume was 5 μL. MS analysis was performed in the positive ion mode. The instrument was calibrated using an external standard before analysis to ensure a mass accuracy better than 3 ppm throughout the experiment. The HESI source parameters were as follows: spray voltage at 3.5 kV, capillary temperature at 320 °C, sheath gas flow rate at 30 arb. units, aux gas flow rate at 10 arb. units, sweep gas flow rate at 5 arb. units, heater temperature at 350 °C, S-lens RF level at 55. A full MS scan (*m*/*z* 70–1000) with a resolution of 70,000 was used. A total of 3 scans per second could be obtained, which provided sufficient data points for quantification. The MS2 scan used a normalized collision energy of 35 V, an isolation window of 0.8 *m*/*z*, and a mass resolution of 35,000. The total phenolic content of CHC-Herb was assayed with the method reported by Taga et al. [37]. Gallic acid was set as the standard. The results were expressed as mg gallic acid equivalents (GAE)/g extract. Total flavonoid content was quantified calorimetrically using the method reported by Jia et al. [38]. Catechin was used as the standard. Data were expressed as mg catechin equivalents (CE)/g extract.

Based on the above assay, we designed a cocktail to mimic CHC-Herb (PF-Cocktail) in order to simplify the bioactive components of CHC-Herb. The PF-Cocktail contained phenolic acids and flavonoids identified with UPLC in CHC-Herb (details in Results). The PF-Cocktail was used for mouse gavage trials and for the treatment of primary chicken hepatocytes.

### 2.3. Assay of Absorption Ability of CHC In Vitro

CHC efficacy in absorbing deoxynivalenol (DON), aflatoxin B1 (AFB1), ochratoxin A (OTA), and zearalenone (ZEN) was evaluated in phosphate buffered solution (0.01 M PBS, pH 2.0 or pH 6.0) and simulated gastric fluid (0.2% NaCl, 0.2% pepsin, pH 2.0), according to the methods of Avantaggiato et al. [39]. Briefly, CHC (10 mg) was suspended with PBS or simulated gastric fluid (10 mL) containing mycotoxins. The concentration ranges of individual mycotoxins were as follows: DON (0.5, 1, 5, 10 μg/mL), AFB1 (10, 50, 100, 200 ng/mL), ZEN (0.2, 0.5, 2, 5 μg/mL), and OTA (50, 100, 200, 500 ng/mL), respectively. The suspensions were mixed and shaken for 90 min at 37 °C and 150 rpm. Then, the supernatants were collected by centrifugation at 18,000× *g* for 4 min at 25 °C. The supernatants were analyzed for residual mycotoxin content. For an assay of the desorption rate, the pellets from absorption assays were completely removed by washing with 10 mL of PBS at pH 8.0 and then shaken for 24 h at 37 °C and 150 rpm. The supernatants were collected for mycotoxin measurement. The Mycotoxins DON, AFB1, OTA, and ZEN were analyzed with Ultra Performance Liquid Chromatography (UPLC) [40]. The pretreated sample was centrifuged, and the supernatant was divided into two aliquots. The first aliquot (used in the aflatoxins analysis) was cleaned up with the use of immunoaffinity columns (AflaTest^®^, Vicam, Waters, Milford, MA, USA). The second, used for the analysis of other mycotoxins, was cleaned up with the use of C18 sorbent (50 mg) and magnesium sulphate (150 mg). Extracts were evaporated, mixed with a labelled internal standard solution (used for quantitation), and determined using a UPLC-MS/MS technique. The LC-MS/MS analysis of all the targets was carried out by using the Waters ACQUITY UPLC system (Waters, Milford, MA, USA) coupled to a 5500 Quadrupole TRAP^®^ hybrid triple quadrupole/linear ion trap MS (AB SCIEX, Foster City, CA, USA) via a Turbo V Ion Spray interface with an electrospray ionization (ESI) source. Analyst^®^ 1.6.2 software (AB SCIEX) was used to control the UPLC-QTrap-MS/MS system and for data acquisition and processing. Nitrogen was used as the nebulizer (GS1), heater (GS2), and curtain (CUR) gas as well as the collision activation dissociation gas. Each mycotoxin standard was directly infused into the mass spectrometer to obtain the MS/MS parameters for each analyte. The MS/MS was performed with electrospray ionization (ESI) in positive mode under the multiple reaction monitoring (MRM) condition. Ion source temperature was set at 550 °C, and the spray voltage was +5500 V. Ion sources GS1 and GS2 together with CUR gas were set at 55, 55, and 35 psi, respectively. The MRM mode was used for quantitation. The UPLC separation of all analytes was carried out on a SHISEIDO Capcell Core C18 column (2.1 × 50 mm, 2.7 μm) at a flow rate of 0.3 mL/min. The mobile phase was composed of 0.1% formic acid aqueous solution (A) and ACN with 0.1% formic acid (B) using the following gradient elution program: 0–2.0 min, linear change from 25 to 55% B; 2.0–5.5 min, from 55 to 90% B; 5.5–5.51 min, switch from 90 to 25% B, and hold at 25% B for an additional 2 min to re-equilibrate the column. The column was kept at 30 °C, and the injection volume was 2 μL. All trials were performed in triplicate. Absorption and desorption values were calculated for each toxin and expressed as percentages.

### 2.4. Antioxidant Activities of CHC In Vitro

The radical scavenging activity of CHC against 2,2-diphenyl-1-picrylhydrazyl (DPPH) was measured according to Shimada et al. [41]. Various concentrations (25, 50, 100, and 150 μg/mL) of CHC were subjected to the assay. Vitamin C was used for comparison. DPPH radical scavenging activity was determined by measuring the absorbance at 517 nm (OD_517_). Each sample was repeated three times. A blank control that included all reagents without CHC (OD_517-blank_) was used to correct readings for the samples. The scavenging activity of the DPPH radical was calculated as follows: [(1 − OD_517_)/OD_517-blank_] × 100%. Total antioxidant activities of CHC were determined using the ABTS (2,2′-azino-bis(3-ethylbenzthiazoline-6-sulfonic acid radical cation) assay [42]. Trolox was used for comparison. Total antioxidant activity was determined by measuring the absorbance at 734 nm (OD_734_). A blank control that included all reagents without CHC (OD_734-blank_) was used to correct readings for the samples. Each sample was repeated three times. The total antioxidant activity was calculated as follows: [(1 − OD_734_)/OD_734-blank_] × 100%. Superoxide radical scavenging activity was determined according to procedures reported by Jing and Zhao [43]. Absorbance was measured at 420 nm (OD_420_). Vitamin C was used for comparison (OD_420-VC_). A blank was the absorbance of Tris-HCl buffer instead of the pyrogallol solution (OD_420-blank_). Each sample was repeated three times. The superoxide radical scavenging activity was calculated as follows: [1 − (OD_420_ − OD_420-blank_)/OD_420-VC_] × 100%.

### 2.5. Cell Culture and Treatments

To reveal the anti-inflammatory effect of CHC-Herb in vitro, the RAW 264.7 cell line was cultured in Dulbecco’s modified Eagle’s medium (DMEM) supplemented with 10% heat-inactivated fetal bovine serum, 100 units/mL penicillin, and 0.1 mg/mL streptomycin and then incubated at 37 °C, in an atmosphere of 85 % humidified air with 5% CO_2_. The reagents were all from Gibco, Thermo Fisher Scientific, Grand Island, NY, USA. The cells were plated into 24-well plates at a concentration of 10^6^ cells/mL and with a final volume of 1 mL. Cell viability was measured after 24 h of incubation with CHC-Herb, using the Vybrant 3-(4,5-dimethylthiazol-2-yl)-2,5-diphenyltetrazolium bromide (MTT) cell proliferation assay kit (Thermo Fisher Scientific, Grand Island, NY, USA). Cells were pretreated with various concentrations of CHC-Herb for 2 h and then treated with lipopolysaccharide (LPS, 1 μg/mL, Sigma-Aldrich, St. Louis, MO, USA) for an additional 24 h. The supernatants were collected and analyzed to obtain the levels of nitric oxide (NO, Promega Corp., Madison, WI, USA) and IL-6 (Bio-Rad Laboratories Inc., Irvine, CA, USA) using commercial kits, according to the manufacturers’ instructions. The control was the DMEM medium without LPS or CHC-Herb. L-N-methylarginine (NMMA, 100 μm) was used as comparison.

To reveal the effects of the PF-Cocktail on gene expression, hepatocytes from adult broiler livers (180–250 g) were isolated by collagenase perfusion in situ [44] and purified by centrifugation by applying Percoll for better separation [45]. Isolated hepatocytes were cultured with M199 in 35 mm dishes. The dishes were coated with collagen prepared from rat tail tendons (1 g/300 mL), which had been dissolved in 0.1% acetic acid for 24 h. The M199 culture medium additionally contained 4% new-born calf serum, 15 mM Hepes, 10 mM glucose, 0.2% BSA, and 10−7 M insulin. Every dish was filled with 10^6^ cells/mL and 2 mL of the culture medium. The medium was changed after 4 h and 24 h. The hepatocytes were cultured for 48 h in a gas atmosphere containing 5% CO_2_ at 37 °C. The cells were incubated with phenolic acids (PAs, composed of caffeic acid and vanillin at a ratio of 1:1, *w*/*w*; 150 μg/mL), flavonoids (FVs, composed of daidzein and quercetin-D-glucoside at a ratio of 1:1, *w*/*w*, 50 μg/mL), PF-Cocktail (PAs+FVs, 200 μg/mL), and CHC-Herb (200 μg/mL) for 48 h before being harvested for mRNA quantification. All the reagents above in the cell culture were purchased from Cayman (Cayman Chemical, Ann Arbor, MI, USA).

### 2.6. Animals, Diets, and Experimental Design

Broiler feeding studies were conducted via two experiments. Experiment 1 (Exp. 1) was a dose-dependent trial that aimed to find a responsive dose for the following experiment. A total of 540 male Arbor Acres chicks (42.8 ± 0.7 g body weight, BW) were randomly assigned to 5 dietary treatments (6 pens/treatment; 18 birds/pen): a corn-soybean meal basal diet (CON) and CON supplemented with 250, 500, 750, 1000 mg/kg of CHC (CHC250, CHC500, CHC750, CHC1000, respectively). Experiment 2 (Exp. 2) was carried out to compare the CHC effects with its constituents, herb extracts, and activated charcoal. A total of 432 chickens (45.1 g ± 1.0 g BW) were randomly assigned to 4 dietary treatments: CON, CON supplemented with 25 mg/kg of CHC-Herb (Herb), CON supplemented with 225 mg/kg of activated charcoal (AC), and CON supplemented with 250 mg/kg of CHC (CHC). The 250 mg/kg dose of CHC was responsive to the CHC treatment from Experiment 1. For preparation of CHC, herb extracts were mixed with activated charcoal at a ratio of 1:9 (*w*/*w*), which meant herb extracts took up of 10% of CHC weight, and activated charcoal took up 90% of CHC weight. Thus, in broiler diets, the supplemental doses of herb extracts and activated charcoal were 25 mg/kg and 225 mg/kg, respectively. All chicks were given ad libitum access to feed and water for 42 days. The diets were offered in two phases (starter phase: d 1–21; grower phase: d 22–42. Table 1). Room temperature was maintained at 34~35 °C during the first 7 d and was then gradually reduced to 25~26 °C, with a drop of 2 °C per week. The broilers were vaccinated against Newcastle on d 7 and against infectious bursal disease on d 14. The corn-soybean meal basal diet met the nutrient requirements of broilers, as stated by NRC [46]. All diets were prepared in one batch. Herb extracts, activated charcoal, or CHC was first mixed with premixed vitamins and minerals, subsequently mixed with other ingredients, and then stored in covered containers. All diets were made in one batch and stored in a cool and dry environment. A fresh feed sample (from same source and batch) without moistening or the addition of additives was kept at −20 °C until the samples were shipped for mycotoxin concentration analyses. All the feed samples were analyzed in order to determine the dietary mycotoxin concentration before being administered to experimental broilers.

This experiment was carried out at the National Feed Engineering Technology Research Center of the Ministry of Agriculture Feed Industry Center Animal Testing Base (Hebei, China). All procedures used in this study were conducted in accordance with Chinese Guidelines for Animal Welfare and approved by the China Agricultural University Institutional Animal Care and Use Committee (AW52501102-1, AW54906203).

### 2.7. Growth Performance and Sample Collection

The body weight of birds was recorded on days 1, 21, and 42 of the experiment after a 12 h withdrawal of feed, but not water. Average daily body weight gain (ADG) was calculated for the starter phase (d 1–21), grower phase (d 21–42), and whole phase (d 1–42). Average daily feed intake (ADFI) was recorded. On days 21 and 42, one broiler closest to the average BW of each pen was selected for blood sampling from the jugular vein. On d 42, the birds were euthanized after blood collection. Intestine tissues were sampled for histological analysis. In Experiment 2, mucosa from intestines was scraped using glass slides, and the liver and kidneys were sampled, chilled, and stored at −80 °C for further analysis. Sampled breast and thigh muscles were stored at 4 °C for meat quality assessment and at −80 °C for further analysis.

### 2.8. Meat Quality Assessment

Immediately following fabrication, boneless and skinless breast and thigh samples from the right side were assessed for pH and meat color. Muscle pH at 45 min and 24 h after slaughter (pH_45min_ and pH_24h_) were determined in triplicate by a portable pH meter (Testo 205, Melrose, MA, USA) [47]. Lightness (L*), redness (a*), and yellowness (b*) values were determined at 24 h postmortem with a spectrometer (CM-3500d; Konica Minolta, Tokyo, Japan), according to the methods of Li et al. [48]. Shear force was measured using a TA.XT plus texture analyzer (Stable Micro Systems Ltd., Surrey, UK), with a 5 kg load cell and a Meullenet-Owens Razor Shear Blade. The water holding capacity (WHC) of meat was assayed according to the methods of Wardlaw et al. [49]. Every measurement was performed in triplicate. The amount of protein in the breast and thigh meat was calculated using the method 981.10 described by the Association of Analytical Chemists [50].

### 2.9. Analyses of Indices of Antioxidant Enzymes, Inflammatory and Immune Factors of Broilers

The activity of serum and tissue malondialdehyde (MDA), total superoxide dismutase (T-SOD), and indices of interleukin-1β (IL-1β), interferon-γ (IFN-γ), and insulin-like growth factor 1 (IGF-I) were measured using commercial assay kits (Nanjing Jiancheng Bioengineering Institute, Nanjing, China), according to manufacturers’ instructions. Intestinal secretory immunoglobulin (SIgA) concentrations were assayed using an Sn-69513-type immune counter (Shanghai Nuclear Annular Photoelectric Instrument Co., Ltd., Shanghai, China). The analysis of serum triglyceride, total cholesterol, low-density lipoprotein cholesterol, and high-density lipoprotein cholesterol were performed using an automatic biochemical analyzer (Hitachi 7600, Tokyo, Japan).

### 2.10. Quantitative Analyses of Bile Acids

Bile acids were quantified using our established methods [51], with modifications. Briefly, tissues were weighed, and internal standards were added before extraction with 0.2 M NaOH (0.5–6 mL) at –80 °C for 20 min. After cooling, 1.5–6 mL of water was added, and the samples were purified using liquid–liquid extraction with 1.5–6 mL of hexane. The extraction step was repeated three times, and the water phases were combined and the samples were further purified with Oasis HLB 3 cc 60 mg (Waters, Milford, MA, USA). There were 6 internal standards (D4-glycocholic acid (GCA), D4-glycodeoxychlic acid (GDCA), D4-cholic acid (CA), D4-Ursodeoxycholic acid (UDCA), D4-lithocholic acid (LCA), and D4-glycochenodeoxycholic acid (GCDCA); 50 nM for each). All mixtures were kept at –20 °C for 10 min and were then centrifuged at 13,000× *g* and 4 °C for 15 min. A 240 μL aliquot of the supernatant was transferred and vacuum dried. A total of 40 μL acetonitrile-methanol (9:1, *v*/*v*) containing 0.01% formic acid was added. The supernatant from the extraction was used for UPLC-MS analysis. A Waters ACQUITY ultra performance LC system equipped with a binary solvent delivery manager and a sample manager (Waters, Milford, MA, USA) was used for BA analysis. The mass spectrometer was a Waters XEVO TQ instrument with an ESI source (Waters Corp., Milford, MA, USA). The entire LC−MS system was controlled using the MassLynx software (Version 4.1). All chromatographic separations were performed with an ACQUITY BEH C18 column (1.7 μm, 100 mm × 2.1 mm internal dimensions) (Waters Corp., Milford, MA, USA). The mobile phase consisted of water with 0.01% formic acid (mobile phase A) and acetonitrile/methanol (9/1, *v*/*v*) with 0.01% formic acid (mobile phase B).

### 2.11. Preparation of Total RNA and Quantitative Reverse Transcriptase PCR (qPCR)

Total RNA was isolated using the RNAgents Total RNA Isolation System (Promega, Madison, WI, USA). RNA samples were reverse transcribed in a Programmable Thermal Controller (PTC)-100 (MJ Research, Watertown, MA, USA). RNA was converted into cDNA using the commercial kits (all from Promega). The primers of the qPCR analysis were synthesized in Sangon Biotech (Shanghai, China). The quantitative real-time PCR reaction was performed with the PowerUp SYBR Green PCR Master Mix (Applied Biosystems, Thermo Fisher Scientific, San Jose, CA, USA), and the reaction was accomplished using the QuantStudio 7 Flex Real-Time System (Applied Biosystems Instruments, Thermo Fisher Scientific, San Jose, CA, USA). The gene expression values were normalized in terms of the glyceraldehyde-3-phophate dehydrogenase (GAPDH) levels and were expressed as fold changes relative to the control group by using the 2^−ΔΔCT^ method. The primers of CYP450 isozymes, metabolism enzymes, nuclear receptors, and reference gene GAPDH were designed using Primer Premier 5.0 and are listed in Table 2.

### 2.12. Assessment of Efficacy of PF-Cocktail for Acute T-2 Mycotoxin Challenge

Male ICR mice (21–24 g) (Weitonglihua Limited Co., Beijing, China) were maintained at a controlled temperature (~22 °C) and had ad libitum access to feed and water. The T-2 mycotoxin challenge was performed according to a reported method [52]. The T-2 toxin stock solution (Cayman Chemical, Ann Arbor, MI, USA) was diluted with a solution of propylene glycol and ethanol (mixed ratio was 9:1, *v*/*v*), and 100 μL of diluted T-2 toxin was administered to mice by subcutaneous injection (2 mg/kg). After 1 h, the mice were assigned to 4 different treatments: saline (CON), activated charcoal (AC, 7 g/kg BW), PF-Cocktail and AC (PF-Cocktail+AC, 0.78 g/kg BW of PF-Cocktail and 7 g/g BW of activated charcoal), and CHC (7 g/kg BW). Mice were gavaged with saline, activated charcoal, PF-Cocktail, respectively. All experiments were performed on 16 h fasted mice. The mice were free to feed 2 h after treatment. The number of surviving mice for each treatment (*n* = 10) was determined at different times after exposure to T-2 mycotoxin. Data were analyzed for statistical significance using the Graphpad Prism 7 software. All procedures used in this study were conducted in accordance with the Chinese Guidelines for Animal Welfare and approved by the China Agricultural University Institutional Animal Care and Use Committee (MC3965881).

### 2.13. Statistical Analysis

Differences among the treatments were determined using the Student *t*-test or a one-way analysis of variance (ANOVA) with the JMP software program (JMP^®^, version 14; SAS Institute Inc., Cary, NC, USA). Mean comparisons were conducted using Duncan’s New Multiple Range Test. The errors were presented as Standard Errors of Mean (SEM). Regression analysis was used to test the linear and quadratic effects of increasing levels of CHC supplementation. Differences were regarded as statistically significant at *p* < 0.05; an indicative trend was defined as 0.05 ≤ *p* < 0.10. Survival analyses were conducted using the Log-rank (Mantel–Cox) test in GraphPad Prism 7.

## 3. Results

### 3.1. Characterization of CHC-Herb

**Figure 1:**
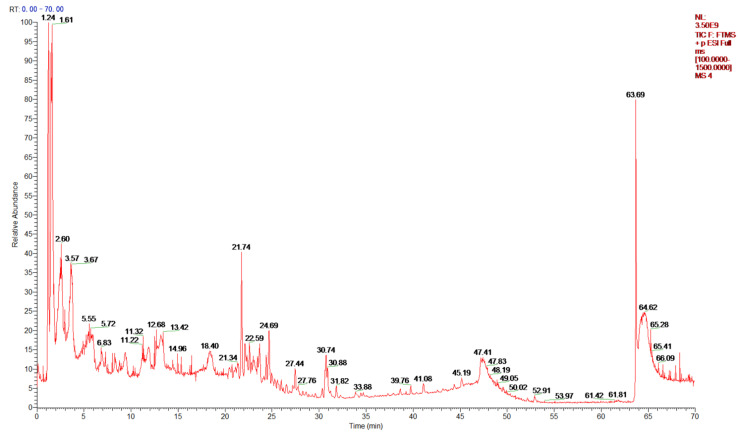
UPLC chromatogram of CHC-Herb.

CHC-Herb contained various phytochemical compounds. The level of total phenolic compounds was 110.4 ± 2.2 mg of equivalent gallic acid per gram of extract. The flavonoid concentration was 40.9 ± 0.8 mg of equivalent catechin per gram of extract. CHC-Herb was analyzed with UPLC (Figure 1), and the active components are presented in Table 3 according to their abundance, as reflected by the peak areas. The active components of CHC-Herb include phenolic acids (salsolinol, shogaol, caffeic acid, vanillin), flavonoids (daidzein, formononetin, puerarin, quercetin-3-β-glucodide, calycosin, kaempferol-7-β-D-glucopyranoside, astragalin, isorhamnetin-3-glucoside, genistein), and organic acids (citric acid, azelaic acid, succinic acid, isocitric acid). To dissect the function of the bioactive components of CHC-Herb, we designed a mixture of phenolic acids and flavonoids called PF-Cocktail in order to mimic CHC-Herb based on the quantification assay, for further studies. The PF-Cocktail contained 110.4 mg of phenolic acids (PAs, composed of caffeic acid and vanillin at a ratio of 1:1, *w*/*w*) and 40.9 mg of flavonoids (FVs, composed of daidzein and quercetin-D-glucoside at a ratio of 1:1, *w*/*w*) per gram of PF-Cocktail solution (1 g/mL). In simple words, caffeic acid and vanillin (representing PAs) along with daidzein and quercetin-D-glucoside (representing FVs) were used to represent the herb components of CHC-Herb.

### 3.2. In Vitro Assay of CHC and CHC-Herb Activities

**Figure 2:**
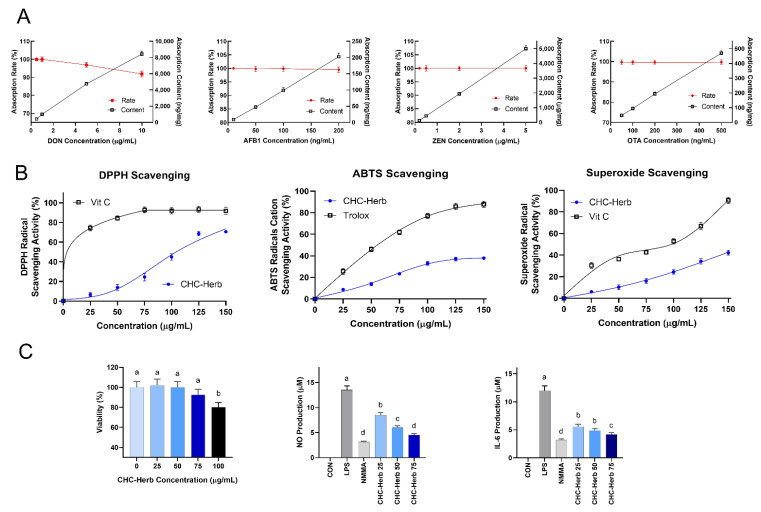
In vitro evaluation of CHC and CHC-Herb activity. CHC absorption rate and absorption of mycotoxin content (DON, AFB1, ZEN, and OTA) (**A**); CHC-Herb scavenging free radicals (DPPH radical, ABTS radical cation, and superoxide radicals) (**B**); and CHC-Herb antioxidative effects in the inhibition of NO production and secretion of anti-inflammatory factor, IL-6, in RAW 264.7 cells (**C**). DON, deoxynivalenol; AFB1, aflatoxin B1; ZEN, zearalenone; OTA, ochratoxin A. Herb, extracts of herbal part of CHC. Vit C, vitamin C. Results are expressed as mean ± standard deviation. Each assay was conducted in triplicate. Cells were pretreated with various concentrations of CHC-Herb for 2 h and then treated with LPS (1 μg/mL) for an additional 24 h. CHC-Herb 25, CHC-Herb 50, CHC-Herb 75, and CHC-Herb 100 represent supplemented doses of CHC-Herb at 25, 50, 75, and 100 μg/mL in cultured medium. Control was DMEM medium, without LPS or CHC-Herb. NMMA (100 μm) was a comparison with CHC-Herb. LPS, Lipopolysaccharide. NMMA, L-N-methylarginine. Bars with different letters represent statistical differences (*p* < 0.05).

As the concentrations of mycotoxin increased in the solutions (DON, AFB1, OTA, and ZEN), the absorption of mycotoxin content was progressively increased (Figure 2A). When the DON concentrations were within 1 μg/mL, CHC absorption rates were 100%; when the DON concentrations were higher than 10 μg/mL, the absorption rates dropped to 91.92 ± 0.81%, with the absorption content at 8.432 μg/mg. When the AFB1 concentrations ranged from 10 to 200 ng/mL, CHC absorption rates were within 99.48 ± 0.89 and 100 ± 0.92%. When the ZEN concentrations were 5 μg/mL or lower, the absorption rate was maintained at 100 ± 0.86%. When the OTA concentrations were 500 ng/mL or lower, the absorption rates ranged from 99.63 ± 0.90 to 99.74 ± 0.85%, with the absorbed content at 469.44 ng/mg. In the PBS solutions(pH 2.0 and pH 6.0), andin gastric solutions (data not shown), CHC maintained its high absorption rates for AFB1, ZEN, and OTA, which ranged from 95.78 ± 0.86 % to 100 ± 0.01%, while CHC’s absorption rate for DON in simulated gastric solutions (69.34 ± 0.78%) was lower than it was for AFB1 (100 ± 0.01%), ZEN (99.23 ± 0.04%), and OTA (95.78 ± 0.86%). The desorption rate of CHC for mycotoxins were as low as 0.00 ± 0.01% to 0.52 ± 0.07%.

As the concentrations of CHC-Herb increased, the DPPH scavenging effects increased (Figure 2B). The SC_50_ of the DPPH radical scavenging activity of CHC-Herb was 89.5 μg/mL. It showed 70% DPPH radical scavenging ability at 150 mg/mL, which was lower than the effect of vitamin C. CHC-Herb (150 μg/mL) showed a total antioxidant activity of 38%, while it was 89% in comparison to Trolox. The SC_50_ of the *ABTS* radical cation scavenging activity of CHC-Herb was 65.1 μg/mL. In the present conditions, CHC-Herb showed lower free radical scavenging activities as compared to Trolox when scavenging ABTS radical cation. At a concentration of 150 μg/mL, the scavenging activity of CHC for superoxide was 40.9% as compared to 93.2% for vitamin C. The SC_50_ of the superoxide radical scavenging activity of CHC-Herb was 88.1 μg/mL.

To determine the cytotoxic potential of CHC-Herb, RAW 264.7 cells were treated with different concentrations of CHC-Herb, and cell viability was measured after 24 h of incubation (Figure 2C). The results showed no significant cytotoxicity in the RAW 264.7 cells at their effective concentrations (≤75 μg/mL). Therefore, the maximum concentration of CHC-Herb was limited to 75 μg/mL. The production of IL-6 by the cells was considerably reduced by the treatment with CHC-Herb (25, 50, and 75 μg/mL) as compared with the control. CHC-Herb at these concentrations inhibited LPS-induced NO production in the RAW 264.7 cells. The results demonstrated the anti-inflammatory and anti-oxidative effects of CHC-Herb in vitro.

### 3.3. CHC Effects on Growth Performance of Broilers

**Figure 3:**
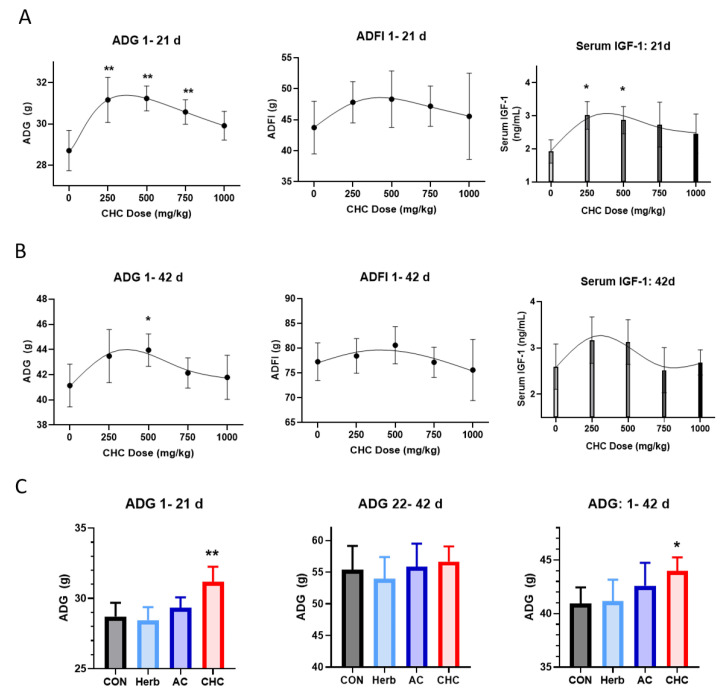
Effects of dietary supplementation on growth performance of broilers. In Experiment 1 (**A**,**B**), different doses of CHC (0, 250, 500, 750, and 1000 mg/kg) were supplemented in corn-soybean-based diets, which were fed to broilers for 42 days. In Experiment 2 (**C**), broilers were fed CON, Herb (25 mg/kg), AC (225 mg/kg), or CHC (250 mg/kg)-supplemented diets for 42 days. CON was corn-soybean basal die. Herb, herb extracts. AC, activated charcoal. ADG, average daily gain; ADFI, average daily feed intake; IGF-1: insulin-like growth factor 1. *t*-test was performed to compare differences between CON and the treatment group. Significant differences were labelled as ** (*p* < 0.01) or * (*p* < 0.05).

In Experiment 1, during d 1–21, ADG was increased in the broilers treated with 250 or 500 mg/kg as compared to the unsupplemented birds fed with CON (*p* < 0.05, Figure 3A). As the supplementation level of CHC increased, ADG tended to responded quadratically (*p* = 0.042) on d 21. During d 1–42, CHC supplementation at 500 mg/kg increased ADG (*p* = 0.035) (Figure 3B). Accordingly, serum IGF-1 showed a well-matched trend to ADG values at d 21 and d 42. CHC showed its active effect on ADG at the supplemental dose of 250 mg/kg; thus, CHC at 250 mg/kg was selected for the comparison with its two constituents, Herb and AC, for the following study on broiler growth. In Experiment 2, Herb (25 mg/kg) and AC (225 mg/kg) supplementation did not show any improvement of ADG during d 1–21 and d 1–42 as compared to CON (Figure 3C). These results indicate a synergy of Herb and AC combined with CHC in the promotion of broiler growth performance.

### 3.4. CHC Effects on Antioxidative and Inflammatory Indices of Serum and Tissues

**Figure 4:**
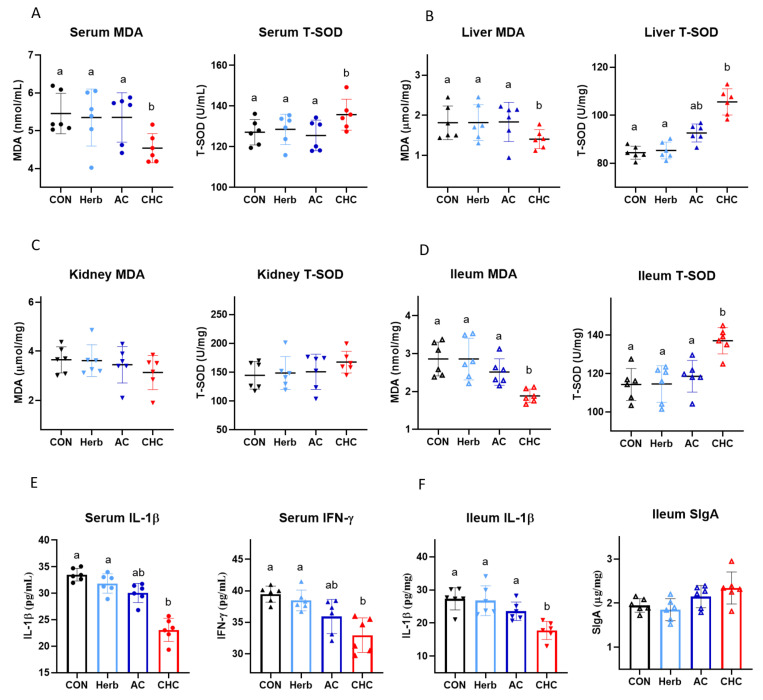
CHC effects on antioxidative and inflammatory indices in broilers. The indices include antioxidant indices (MDA and T-SOD) in the serum (**A**), liver (**B**), kidney (**C**), and ileum (**D**); anti-inflammatory and immune indices (IL-1β, IFN-γ, and SIgA) in the serum (**E**) and ileum (**F**) on d 21. Broilers were fed with CON, Herb (25 mg/kg), AC (225 mg/kg), or CHC (250 mg/kg)-supplemented diets for 42 days. CON was corn-soybean basal die. Herb, herb extracts. AC, activated charcoal. One-way ANOVA was performed for statistical analysis. Bars with different letters indicate significant differences (*p* < 0.01).

In CHC-treated broilers, MDA levels were decreased and T-SOD activities were increased in the serum, liver, and ileum (Figure 4A,B,D. *p* < 0.01), except for the kidney (Figure 4C), as compared to CON. Levels of MDA or T-SOD as compared to that of the CON group were not significantly altered by the sole supplementation of Herb or AC in the diets. CHC supplementation decreased the IL-1β and IFN-γ levels in the serum and the IL-1β level in the ileum as compared to CON (Figure 3E,F. *p* < 0.01). Similar to the indices of antioxidants, only CHC (not Herb or AC) altered IL-1β and IFN-γ levels in the serum and ileum.

### 3.5. CHC Effects on Meat Quality of Broilers

Dietary treatments had no effects on the pH_45min_ or pH_24h_ of the breast and thigh muscles of broilers (Table 4). The water-holding capacity at 24 h was not affected by dietary treatments. Regarding meat color, CHC decreased the lightness of the breast muscle (*p* < 0.05), while it increased the redness of the thigh muscle (a*, *p* < 0.05). CHC tended to decrease the shear force of the breast (*p* = 0.094) and thigh (*p* = 0.063). Herb and AC showed no significant effects on meat color or shear force.

### 3.6. Effects on Bile Acid Synthesis and Metabolism

**Figure 5:**
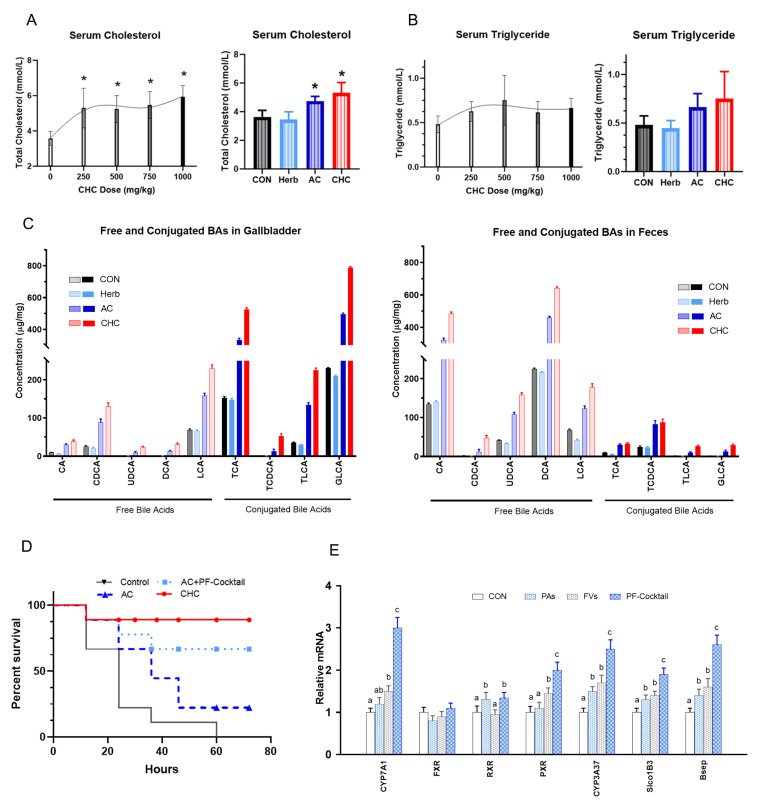
CHC effects on bile acid synthesis, metabolism, and alleviation of liver damage. Effects of CHC supplementation at different doses (0, 250, 500, 750, and 1000 mg/kg), of Herb (25 mg/kg), and of AC (225 mg/kg) on the levels of serum cholesterol (* indicates *p* < 0.05) (**A**) and triglyceride (**B**). Broilers were fed with treatment diets. CON was corn-soybean basal die. Herb, herb extracts. AC, activated charcoal. Specific bile acids in the gallbladder and feces (**C**), including free and conjugated bile acids. Survival rate of four groups of mice gavaged with AC, PF-Cocktail, and CHC upon challenge with oral T-2 mycotoxin (**D**). Male ICR mice were challenged with mycotoxin T-2 by subcutaneous injection (2 mg/kg). After 1 h, these mice were treated with gavage of saline (CON), activated charcoal (AC, 7 g/kg BW), PF-Cocktail and AC (PF-Cocktail+AC, 0.78 g/kg BW of PF-Cocktail and 7 g/g BW of activated charcoal), and CHC (7 g/kg BW), respectively. The number of surviving mice for each treatment (*n* = 10) was determined at different times after exposure to T-2 mycotoxin. Survival analyses were conducted using the log-rank (Mantel–Cox) test in GraphPad Prism 7. Treatments with phenolic acids (PAs), flavonoids (FVs) or PF-Cocktail altered the expression of xenobiotic detoxification enzymes in broiler primary hepatocytes (**E**). The mRNA levels of metabolic enzymes and nuclear receptors involved in bile synthesis, metabolism, and xenobiotic detoxification (*CYP7A1, FXR, RXR, PXR, CYP3A37, Slco1B3, and Bsep*) in cultured primary chicken hepatocytes were quantified by qRT-PCR, with GAPDH mRNA as an internal control. PAs (phenolic acids, composed of caffeic acid and vanillin at a ratio of 1:1, *w*/*w*, 150 μg/mL); FVs (flavonoids, composed of daidzein and quercetin-D-glucoside at a ratio of 1:1, *w*/*w*, 50 μg/mL); PF-Cocktail (PAs+FVs, 200 μg/mL). One-way ANOVA was performed for statistical analysis. Bars with different letters indicate significant differences within a quantified gene (*p* < 0.01).

Supplementation with CHC at doses of 250, 500, 750, and 1000 mg/kg increased the serum levels of cholesterol as compared to the unsupplemented CON (Figure 5A, *p* < 0.01). However, as the dose of CHC increased, the cholesterol levels did not increase accordingly (Figure 5B, *p* > 0.05), which indicated a path for cholesterol excretion. Both CHC and AC which had similar dosed AC as CHC contained, increased serum cholesterol as compared to CON (*p* < 0.01). CHC and AC supplementation numerically increased the serum levels of triglyceride without any statistical difference (*p* > 0.05). Analysis of bile acids in the gallbladder revealed that supplemented CHC and AC significantly increased free bile acids (cholic acid (CA), chenodeoxycholic acid (CDCA), ursodeoxycholic acid (UDCA), deoxycholic acid (DCA), and lithocholic acid (LCA)) and conjugated bile acids (taurocholic acid (TCA), taurochenodeoxycholic acid (TCDCA), taurolithocholic acid (TLCA), and glycolithocholic acid (GLCA)) as compared to CON (Figure 5C, *p* < 0.01). Both CHC and AC resulted in a greater level of free and conjugated bile acids in feces in comparison to CON, which indicated that CHC and AC promoted the excretion of bile acids from broilers via feces. Comparatively, CHC had much greater effects than AC in the stimulation of bile acid synthesis and excretion (*p* < 0.01).

### 3.7. Effects of PAs and FVs Representing CHC-Herb on Detoxification of T-2 Mycotoxin in Mice

The survival rate of mice in the control (saline) treatment declined steadily throughout the observation period, reaching a value of 10% after 60 h (Figure 5D). All mice treated with AC, AC+PF-Cocktail, or CHC were protected against the lethal effects of T-2 mycotoxin in different degrees (*p* < 0.01). The 50% survival of mice in the control group was significantly lower than the value of 90% for the treated group. The detoxification effect of AC alone was different from the CHC effect (*p* = 0.03), while the effect of AC with the PF-Cocktail showed no difference from that of CHC (*p* = 0.35). These results confirm that CHC had the most significant effects on the detoxification of T-2 toxin. PAs and FVs (PF-Cocktail) represented a majority effect of detoxification of T-2 toxin of the herb portion of CHC.

### 3.8. Effects of PF-Cocktail on Gene Expressions in Primary Chicken Hepatocytes

The genes related to bile acid synthesis, transportation, and metabolism as well as their nuclear receptors that were regulated by PAs, FVs, and the PF-Cocktail were quantified (Figure 5E). The results showed significantly increased mRNA levels of bile acid synthesis (*CYP7A1*), bile salt export pump (*Bsep*), and the genes related to xenobiotic metabolism (*CYP3A37* and *Slco1B3*) due to the PF-Cocktail. Compared to CON, both PAs and FVs alone moderately increased the expression of the above genes, but the increase was much lower than that resulting from their combination (PF-Cocktail). FXR were the target nuclear receptors of bile acids, and bile acids were ligands that activated FXR by conformation alteration. Thus, the expression level of FXR was not significantly altered by the dietary treatments. The PXR expression level was increased by FVs and the PF-Cocktail since it could be regulated by FVs at both the transcriptional level and via conformation alteration. These results proved the synergy of PAs and FVs in the activation of gene expression related to bile acid synthesis and the genes of xenobiotic metabolism.

## 4. Discussion

Mycotoxin contamination has been estimated to affect 25% of global feed production [53]. Concentrations of individual mycotoxins in feed are regulated in many developed countries [54] and in China [55]; however, the regulation of individual mycotoxins ignores the deleterious effects of synergistic interactions among mycotoxins [56]. The severity of mycotoxicosis can be complicated by vitamin deficiency, caloric deprivation, and infectious diseases [57]. Mycotoxins in feed can cause the overproduction of free radicals, which leads to oxidative stress in animals. Oxidative stress and inflammatory reactions are often associated with enteritis in broilers and piglets [58,59].

Dietary supplementation with charcoal or activated charcoal is an effective detoxification strategy due to charcoal’s high absorptive capacity, particularly in relation to a variety of toxins such as mycotoxins, toxic metabolites, and pathogens. Effective doses of activated charcoal in broilers range from 0.3% to 10%, as reported in the literature [11]. In the current study, CHC improved the growth performance of broilers at a very low dose of 250 mg/kg, which is about one-tenth of the reported effective dose for charcoal or activated charcoal alone. In addition, the dietary supplementation of activated charcoal alone at 225 mg/kg, a level similar to that of activated charcoal at 250 mg/kg CHC, did not affect the growth performance of the broilers.

Herb extracts contain various glycosides, phenolics, and flavones, including flavonoids that have intense antioxidant and anti-inflammatory activities [16]. Beneficial effects of plant extracts on poultry performance can be achieved with doses ranging from 0.5% to 3% for mixed extracts [60]. In the current study, we used herb extracts from *Pulsatilla chinensis*, *Portulaca oleracea* L., *Artemisia argyi Folium*, and *Pteris multifida Poir* at a dose as low as 25 mg/kg to supplement diets for broilers. This dosage of herb extracts was similar to the compositional content of herb extracts contained in CHC (10% herb extracts, *w*/*w*). As expected, herb extracts alone at such a low dose had no effect on broiler performance. However, feeding herb extracts at this low dose combined with activated charcoal in CHC improved growth performance and positively affected the anti-inflammatory and antioxidant indices. These findings indicate the cooperative interactions of the charcoal portion and the herb portion of CHC. Consequently, the intrinsic mechanism for the interactions between activated charcoal and herb extracts was investigated.

Firstly, multiple active components in the herb extracts, including total phenolics and flavonoids, were quantified; individual active components were also identified and quantified. In addition to these identified bioactive phenolic acids, flavonoids, and organic acids, there are numerous bioactive compounds that have been reported in the scientific literature (Table S1, refs. [19,20,61,62,63,64,65,66,67,68,69,70,71,72]). Some of these active compounds are ligands of transcription factors for the activation of xenobiotic detoxification genes. For example, flavonoids such as isoflavone, genistein, and daidzein are agonists of the pregnane X receptor (*PXR*) [73]. Phenolic acids such as gallic acid [74], protocatechuic acid [75], chlorogenic acid [76], and caffeic acid [76] are *PXR* ligands that induce *CYP3A4* expression (*CYP3A37* in chicken). *PXR*, a generalized xenobiotic sensor rather than a receptor for endogenous ligands [77], is a master transcription factor of the xenobiotic- and drug-inducible expression of key genes that encode metabolic enzymes and drug transporters [78]. Based on the total phenols, total flavonoids, and the abundance of key phenolic acids and flavonoids in the herb extracts, we designed a cocktail of PAs and FVs representing the active portion of phenols and flavonoids present in CHC’s herb portion to investigate the intrinsic mechanisms in cell culture and in a mouse gavage study.

The herb extracts also contained multiple unsaturated fatty acids. Some unsaturated fatty acids can be ligands in vitro for the activation of the retinoid X receptor (*RXR*). The *RXR* can bridge different signaling pathways by binding with other nuclear receptors as heterodimers [79] such as *FXR* and *PXR*. Unlike steroid receptors that translocate from the cytoplasm to the nucleus once activated by ligands, FXR and PXR are already located in the DNA where they create heterodimers with the RXR. When FXR and PXR’s ligands binding, their structural conformation can change, thus they can interact with co-activator proteins [80]. This may be the reason why gene expression was not significantly changed by the ligands of FXR and PXR in the current study. We measured very low concentrations of fatty acids (4.5 μg per mg of CHC, Table S2, refs. [81,82,83]) in CHC. With such low concentrations of fatty acids, we could not perform the treatment of primary cells. Thus, it is likely that the poly unsaturated fatty acids in CHC did not activate the *RXR*. However, PAs showed a slight enhancement of the *RXR* gene expression, which indicates a possible regulation of caffeic acid and vanillin in *RXR* activity.

Secondly, activated charcoal contained in CHC elevated serum cholesterol, which was the precursor of bile acid under the catalysis of *CYP7A1*. In previous studies, activated charcoal increased cholesterol synthesis in humans [84], increased serum cholesterol in chickens [10] and in piglets [18], but the mechanisms responsible for the elevation of blood cholesterol are not known. Flavonoids such as quercetin, quercetin-glucoside, and kaempferol can elevate hepatic *CYP7A1* expression in mice [85,86,87]. Some phenolic acids increased *CYP7A1* gene expression and increased the concentration of bile acids in feces [88,89]. In the present study, we found the increased expression of *CYP7A1* in primary hepatocytes treated with PAs, FVs, or cocktails of PAs and FVs.

**Figure 6:**
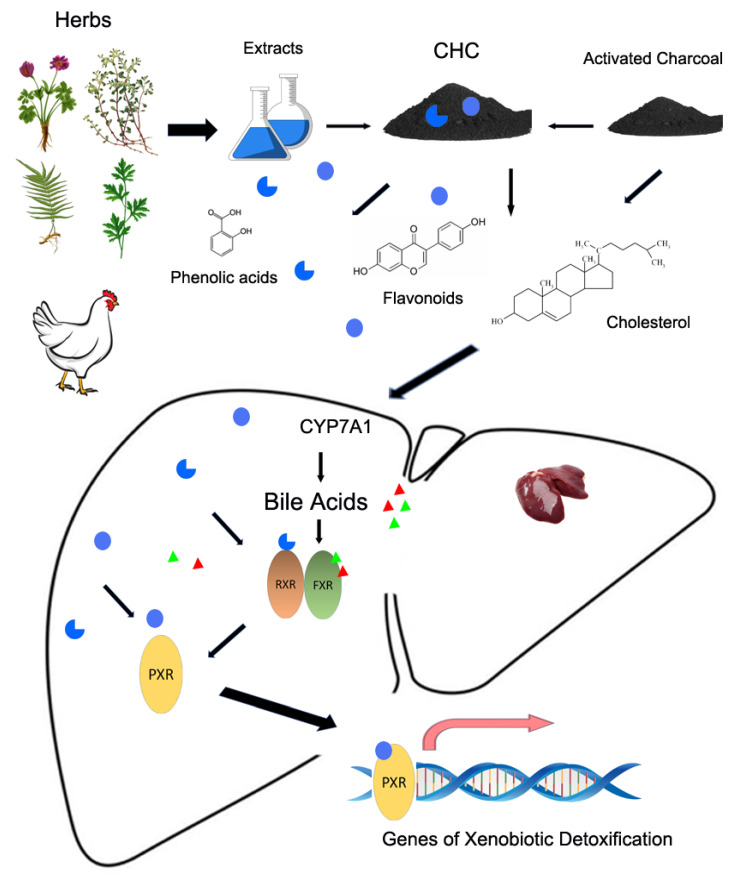
A working model of CHC promotes bile acid metabolism and xenobiotic detoxification in livers of broilers fed with mycotoxin-containing diets. CHC contains two portions: activated charcoal and herb extracts. The portion of activated charcoal can increase cholesterol level, which is the precursor of bile acid synthesis. Phenolic acids and flavonoids increase the synthesis of bile acids such as CDCD, LCA, and CA by enhancing *CYP7A1* expression. These bile acids are the ligands that activate the farnesoid X receptor (*FXR*) transcription factor. The portion of herb extracts contains multiple types of flavonoids and phenols, including daidzein, quercetin, and genistein, which are the ligands that activate the pregnane X receptor (*PXR*), a crucial transcription factor in drug metabolism. Herb extracts also contain numerous types of phenolic acids such as caffeic acid and vanillin, which are potentially active ligands of the retinoid X receptor (*RXR*). *FXR* and *RXR* can form a heterogenous dimer to promote the expression of *PXR*. Subsequently, *PXR* activates the expression of genes for xenobiotic detoxification. Therefore, there are two pathways for the activation of *PXR*: ① Activation of the *FXR-RXR* heterodimer by bile acids and phenolic acids that act on the *PXR* gene promoter, and ② Activation of *PXR* by ligand (flavonoids, phenolic acids, or bile acids) binding. Separately, activated charcoal or herb extract, at a dose similar to that of CHC, is not sufficient to activate *PXR* to trigger the expression of the genes for xenobiotic detoxification. CHC integrates the cooperative interactions between the activated charcoal and the herb extract (CHC) in this detoxification process. Herbs: *Pulsatilla chinensis* (**top left**), *Portulaca oleracea L*. (**top right**), *Artemisia argyi Folium* (**bottom left**), and *Pteris multifida Poir* (**bottom right**). CDCA, chenodeoxycholic acid; LCA, lithocholic acid; CA, cholic acid.

We also found that activated charcoal or activated charcoal contained in CHC had elevated bile acids in the gallbladder and caused a higher excretion of bile acids in feces. CHC elicited a much greater effect than activated charcoal. This indicates a pathway that starts from bile acid synthesis to the clearance of bile acids via feces. This pathway, is elevated by both activated charcoal and the herb extracts. In this process, activated charcoal is responsible for the elevation of cholesterol, while PAs and FVs are responsible for bile acid synthesis and the activation of the transcription factors that could trigger the expression of xenobiotic detoxification genes. Increased bile acids, mainly consisting of lithocholic acid (LCA), chenodeoxycholic acid (CDCA), and cholic acid (CA) in chickens, are active ligands of the farnesoid X receptor (*FXR*). *FXR* is one of the most extensively studied nuclear receptors of bile acids. Upon activation by ligands, *FXR* interacts with its heterodimer partner, retinoid X receptor (*RXR*), and binds to a specific *FXR* response element on the promoter to activate gene expression. A proposed working model that integrates the cooperative interactions of activated charcoal and the herb extracts of CHC is illustrated in Figure 6. In the current study, we used four types of Chinese herbs: *Pulsatilla chinensis*, *Portulaca oleracea* L., *Artemisia argyi Folium*, and *Pteris multifida Poir.* According to the literature and based on their functional components, herb extracts contain phenolic acids and flavonoids that can activate *PXR* at certain concentrations.

Thirdly, we proved that CHC had much higher efficiency in the detoxification of mycotoxin in the mouse gavage study. Mice gavaged with toxic doses of the T-2 mycotoxin were saved by CHC, but activated charcoal or PAs and FVs, separately, were ineffective.

Fourthly, the genes expressed in broiler primary hepatocytes treated with PAs, FVs, or the cocktail (PAs and FVs) were quantified. The genes differentially expressed were related to bile acid synthesis, transportation and metabolism, and the nuclear receptors (transcription factors) that could be activated by PAs or FVs. The pathway of bile acid synthesis, export, and hydrolysis to activate the expression of *PXR*, *FXR,* and *RXR* was revealed. The cocktail of PAs and FVs was especially efficient in enhancing the expression of *CYP7A1* and *PXR* as compared to PAs or FVs alone. This finding indicates t PAs, FVs, or intracellular bile acids are ligands to activate their receptors and transcription factors.

*PXR* has a broad spectrum of ligands [90,91]. *PXR*’s unique features allow it to be activated and mobilized by multiple ligands at much lower concentrations than those required for individual ligands to produce a similar response [92]. In this study, intracellular bile acids that were produced by liver *CYP7A1* may join PAs and FVs as the ligands of *PXR*. Multiple ligands could significantly reduce the required concentration of ligands to activate *PXR*. This was likely the reason why *PXR* was induced by a cocktail of PAs and FVs rather than individual PAs and FVs.

Bile acids can activate *FXR* at physiological concentrations, which bind to *RXR*s forming heterodimers to activate their target gene *PXR*. Therefore, *PXR* could be activated in two pathways. One is via the heterodimer FXR/RXR on the transcriptional level, and the other is via *PXR*’s ligand binding with flavonoids. *FXR* is expressed strongly in the liver and intestine, and it is the master transcriptional regulator of several entero-hepatic metabolic pathways implicated in bile acid, lipid, and glucose homeostasis [93]. Bile acids activate *FXR*, which blocks synthesis and promotes the breakdown of bile acids through *PXR* transcriptional activation, thus making *PXR* the target gene of *FXR* [94]. A key function of the liver is the elimination of xenobiotics and endogenous catabolites from systemic circulation. This elimination generally involves three phases: hydroxylation (phase I), conjugation (phase II), and transport (phase III). As a xenobiotic receptor, PXR regulates many genes involved in detoxification pathways in the liver such as cytochrome P450 (*CYP3A*), *CYP2B*, and *CYP2C* [95]; Phase II conjugation enzymes such as sulfotransferases (SULTs) and Ces 1 [96]; Phase III drug transporters such as OATP2 (in human), Slco1B3 (in chicken), and Bsep [97]. We found that CHC largely increased the expression of these genes of xenobiotic detoxification as compared to a much more moderate effect from activated charcoal and herb extracts. This observation partially proves the cooperative interactions between activated charcoal and bioactive components contained in herb extracts on detoxification pathways. In other words, increased cholesterol levels trigger the initiation of detoxification by increased bile acid levels. Moreover, this effect is amplified by ligand-activated *RXR* and *PXR*. In this way, the toxins in feed are efficiently detoxified by hydroxylation, conjugation, and secretion/excretion.

Besides the components that could activate nuclear receptors upstream of the P450 series enzymes, herb extracts contain other components such as glycosides, polysaccharides, terpenes, and triterpene saponins that have anti-inflammatory activities [20,65,70,72]. In the present study, stimulated macrophages (RAW 264.7 cells) in vitro confirmed the anti-inflammatory activity of the herb extracts of CHC. In vivo, at a low dose of supplementation, the herb extracts showed no significant effect on the stimulation of the anti-inflammatory factors IL-1β and IFN-γ in serum and ileum. However, when the herb extracts were used in combination with activated charcoal, the herb extracts demonstrated very significant anti-inflammatory effects. This effect was an integrated effect because charcoal itself has mild anti-inflammatory effects [15]. Charcoal sorption might protect herb extracts from degradation and conjugation in the intestines [98]. However, this significant anti-inflammatory effect was most likely due to the activation of *PXR*, either by the activated heterodimer *FXR*/*RXR* or the flavonoid ligand, which involves anti-inflammatory processes [99,100].

Mycotoxins and their metabolites can be enzymatically degraded [101]. During this process, free radicals are generated, and oxidative stress arises. When invading pathogens encounter phagocytes, phagocytosis occurs, which induces ROS production in cells [102]. Oxidative stress damages macromolecules such as proteins, DNA, and polyunsaturated fatty acids in membrane lipids. In broilers, oxidative stress can decrease the absorption of nutrients, deteriorate immune capacity, and compromise meat quality. In the current study, CHC alleviated oxidative stress in broilers. One possible mechanism of CHC function might be the absorption of feed-borne toxins. The in vitro assay proved that CHC had a high absorption rate for AFB1, ZEN, and OTA, ranging from 95.78% to 100% at pH values of 2.0 and 6.0 in artificial stomach solutions. Once absorbed, the complex of CHC and mycotoxins was not easily dissociated. In broiler feed, we detected trace contents of DON, ZEN, AFB1, and OTA. In vivo, the antioxidative activity of CHC in broilers was significantly higher than activated charcoal at the same dose. This indicated that CHC’s antioxidative effect was mainly due to the clearance of mycotoxins in accordance with the above pathway.

Oxidative stress is capable of damaging muscle structures and can diminish meat quality of broilers [103]. Markers of meat quality include meat color (lightness, redness, and yellowness), water-holding capacity (drip loss), and tenderness (shear force) [104]. In this study, we found that dietary CHC supplementation increased the antioxidant activity of meat by increasing radical scavenging activities, which in turn decreased lightness in the breast muscle and increased meat redness in the thigh muscle. This result was consistent with broilers fed wood charcoal and vinegar [105]. Poultry meat contains high concentrations of polyunsaturated fatty acids [106], which makes it sensitive to free radical attack and oxidative deterioration [107]. The color of meat is related to the concentration of myoglobin, the chemical state of myoglobin, and light scattering. Myoglobin changes color based on different compounds bound at the iron atom in its protoporphyrin ring structure [108]. When oxygen is bound to the reduced form of molecules, the oxymyoglobin shows its pigment. When no ligand is attached, metmyoglobin forms, which has a purple color. The three chemical states of myoglobin, where iron is oxidized to Fe^3+^ [109], are combined to develop meat color that is acceptable to consumers. Light scatter from denatured proteins or reduced myofilament lattice spacing can also change the lightness of meat [110]. Any factors that help to maintain the integrity of myocytes and myofibrils impact light scattering on the meat surface. CHC’s influence on meat lightness confirms its antioxidant activity. CHC tended to decrease the shear force of the breast and thigh meat in this study.

Taken together, CHC had beneficial effects on broiler growth and meat quality. It alleviated oxidative stress that may have been introduced through feed mycotoxins. The activated charcoal portion increased the cholesterol level, and phenolic acids and flavonoids present in the herb extract portion of CHC enhanced bile acid synthesis by increasing *CYP7A1* expression. The cooperative binding of phenolic acids, flavonoids, and bile acids in hepatocytes activated the *PXR* and *FXR* transcription factors and triggered the expression of xenobiotic detoxification genes through *RXR*. This efficiency of detoxification was proved by CHC’s high rescued rate for mice gavaged with T-2 mycotoxin. CHC synergized the effects of activated charcoal and herb extracts via the *FXR*/*RXR-PXR* pathway to detoxify xenobiotic from the body.

## 5. Conclusions

In conclusion, CHC demonstrated in vitro absorptive activity on mycotoxin, but also antioxidant properties. The in vivo study revealed that CHC integrates the synergy of activated charcoal and herb extracts (CHC) in the detoxification of mycotoxin contained in feed via the promotion of bile acid production and their excretion via feces. At doses similar to those of CHC, activated charcoal or herb extracts fed separately could not act as efficiently as CHC. Activated charcoal, with similar doses of flavonoids (represented by daidzein and quercetin-D-glucoside) and phenolic acids (represented by caffeic acid and vanillin) to those of CHC, functioned no differently from CHC in the detoxification of T-2 mycotoxin in the mouse survival test. Phenolic acids and flavonoids activated the genes for xenobiotic detoxification via the *FXR*/*RXR-PXR* pathway in primary broiler hepatocytes. CHC integrated the functions of flavonoids and phenolic acids contained in the herb extracts and activated charcoal to detoxify mycotoxins from the broiler body.

## Figures and Tables

**Table 1:** Composition and nutrient levels of basal diets (%, dry matter basis).

Item	Starter Phase (d 1–21)	Grower Phase (d 22–42)
Corn	59.85	60.80
Soybean meal	30.13	28.63
Fish meal	4.00	2.77
Soybean oil	2.75	4.54
Dicalcium phosphate	0.88	1.00
Limestone	1.45	1.38
98% DL-Methionine	0.14	0.08
Salt	0.30	0.30
Vitamin-mineral premix ^1^	0.50	0.50
Total	100.00	100.00
Nutrient levels		
Digestible energy, kcal/kg	2222.00	3107.00
Crude protein	21.46	20.00
Calcium	0.99	0.95
Total phosphorus	0.68	0.67
Methionine	0.50	0.41
Lysine	1.13	1.04
Mycotoxin level, measured ^2^		
DON, μg/kg	1146.23	1093.48
AFB1, μg/kg	3.44	4.30
OTA, μg/kg	46.71	50.19
ZEN, μg/kg	237.79	257.65

^1^ The premix provided the following substances per kg of compound feed: vitamin A, 2000 KIU; vitamin D3, 550 KIU; vitamin E, 4000 IU; vitamin K3, 400 mg; vitamin B1, 300 mg; vitamin B2, 1200 mg; vitamin B6, 600 mg; vitamin B12, 2.4 mg; niacin, 6000 mg; pantothenic acid, 2400 mg; folic acid, 60 mg; biotin, 2.4 mg; iron, 18 g; copper, 2 g; zinc, 15 g; manganese, 14 g; iodine, 120 mg; selenium, 66 mg. Nutrient levels, except metabolizable energy, are calculated values. ^2^ Values were measured by UPLC analysis. DON, deoxynivalenol; AFB1, aflatoxin B1; OTA, ochratoxin A; ZEN, zearalenone.

**Table 2:** PCR primer sequences.

Gene	Accession No.	Forward Primers	Reverse Primers
*Bsep*	XM_040676683.2	5′-TGCAAAGCAAAGGAGACT-3′	5′-GCAATGGATAATGGAGGG-3′
*FXR*	XM_046906255.1	5′-AAAGCCGTTCTGTGCGTT-3′	5′-GGATTGGTGGGGTTCCTG-3′
*SLCO1B3*	XM_046939330.1	5′-CAGGACTCTCGCTGGGTGG-3′	5′TGGCTTTCAGGGGCTTTTT-3′
*CYP3A37*	NM_001001751.2	5′-CGAATCCCAGAAATCAGA-3′	5′-AGCCAGGTAACCAAGTGT-3′
*PXR*	NM_204702.1	5′-TCCCTTCGGCATCCCTGTC-3′	5′-GGCGTTGGTCTCCTCGTTG-3′
*RXR*	XM_040686122.2	5′-GATGCGAGACATGCAGATG-3′	3′-GTCGGGGTATTTGTGCTTG-3′
*CYP7A1*	AB109636.1	5′-CATTCTGTTGCCAGGTGATGTT-3′	5′-GCTCTCTCTGTTTCCCGCTTT-3′
*GAPDH*	NM_204305.2	5′-CCATCACAGCCACACAGAAGAC-3′	5′-TGGACGCTGGGATGATGTT-3′

**Table 3:** Bioactive components of CHC-Herb identified with UPLC/MS.

Name	Formula	Annot. DeltaMass (ppm)	Calc. MW	RT (min)	*m*/*z* Vault Best Match	Group Area
Daidzein	C_15_H_10_O_4_	−0.97	254.05766	28.256	90.5	30,805,430.75
Formononetin	C_16_H_12_O_4_	−1.01	268.07329	33.118	92.5	14,380,915.26
Puerarin	C_21_H_20_O_9_	−0.36	416.11058	23.048	80.8	14,368,814.61
Quercetin-3β-D-glucoside	C_21_H_20_O_12_	0.23	464.09558	24.784	87.8	12,538,295.15
Calycosin	C_16_H_12_O_5_	−0.84	284.06823	28.643	79.9	6,191,828.136
Kaempferol-7-*O*-β-D-glucopyranoside	C_21_H_20_O_11_	0.05	448.10059	25.958	85.7	5,872,443.373
Astragalin	C_21_H_20_O_11_	0.63	448.10084	25.745	86.2	5,082,216.64
Isorhamnetin-3-glucoside	C_22_H_22_O_12_	0.52	478.11137	25.912	85.7	3,149,225.104
Genistein	C_15_H_10_O_5_	−0.39	270.05272	30.911	72.7	3,072,532.592
Citric acid	C_6_H_8_O_7_	−0.19	192.02697	3.96	95.8	3,413,221,349
Betaine	C_5_H_11_NO_2_	−0.82	117.07888	1.978	90.1	1,410,250,584
L-(–)-Malic acid	C_4_H_6_O_5_	0.27	134.02156	2.164	97.8	1,071,155,059
Trigonelline	C_7_H_7_NO_2_	0.16	137.0477	2.068	93.6	923,442,478.8
Azelaic acid	C_9_H_16_O_4_	−0.17	188.10483	26.591	93.6	288,761,653.3
Salsolinol	C_10_H_13_NO_2_	−0.2	179.09459	6.145	92.6	251,967,768
Succinic acid	C_4_H_6_O_4_	−0.19	118.02659	4.917	98.1	216,492,622.8
7-Methoxycoumarin	C_10_H_8_O_3_	−0.75	176.04721	25.386	73	76,383,924.11
Isocitric acid	C_6_H_8_O_7_	−0.14	192.02698	2.647	92.2	60,687,891.46
Shogaol6-	C_17_H_24_O_3_	−0.48	276.17241	35.66	83.3	53,880,701.45
Caffeic acid	C_9_H_8_O_4_	−0.3	180.0422	23.338	84.1	49,191,738.9
6,7-Dihydroxycoumarin	C_9_H_6_O_4_	0	178.02661	23.322	87.9	42,206,943.35
Vanillin	C_8_H_8_O_3_	−0.32	152.0473	24.788	83.8	40,006,158.28
2′,4′-Dihydroxy-3,4-dimethoxychalcone	C_17_H_16_O_5_	−1.16	300.09943	39.22		39,938,110.96

**Table 4:** Effects of dietary supplementation on broiler meat quality ^1^.

Item ^2^	CON	Herb	AC	CHC	SD	*p*-Value
Breast						
pH _45min_	6.43	6.41	6.37	6.40	0.45	0.587
pH _24h_	5.58	5.87	5.53	5.92	0.25	0.466
L*	49.94 ^a^	49.61 ^a^	50.22 ^a^	47.91 ^b^	0.76	0.035
a*	2.09	2.23	2.22	2.14	0.09	0.135
b*	7.21	7.35	7.33	7.34	0.07	0.369
WHC, %	78.90	80.36	79.30	81.63	1.72	0.624
Protein, %	23.63	23.80	24.12	24.07	0.51	0.894
Shear force, N	31.95	31.44	31.35	31.26	0.27	0.094
Thigh						
pH _45min_	6.40	6.35	6.45	6.38	0.24	0.669
pH _24h_	6.39	6.85	6.81	6.72	0.16	0.118
L*	69.86	68.91	67.93	68.77	0.89	0.463
a*	3.68 ^b^	4.00 ^ab^	3.61 ^b^	4.13 ^a^	0.18	0.031
b*	6.74	6.72	6.49	6.65	0.16	0.277
WHC, %	73.06	76.18	79.64	77.24	2.86	0.355
Protein, %	17.26	17.53	18.06	17.68	0.44	0.631
Shear force, N	32.65	31.34	31.72	31.03	0.54	0.063

^1^ Values are mean ± standard deviation (SD). Means within the same row without common superscripts differ (*p* < 0.05). ^2^ L*, Lightness. a*, Redness. b*, Yellowness. WHC, water holding capacity. Broilers were fed with CON, Herb (25 mg/kg), AC (225 mg/kg), or CHC (250 mg/kg)-supplemented diets for 42 days. CON was corn-soybean basal die. Herb, herb extracts. AC, activated charcoal. One-way analysis of variance (ANOVA) was performed for statistical analysis. Treatment means were compared using the Duncan’s New Multiple Range Test. Different letters (a,b) indicate significant differences (*p* < 0.05).

## Data Availability

Not applicable.

## Supplementary Materials

The following supporting information can be downloaded at: https://www.mdpi.com/article/10.3390/antiox11112200/s1, Table S1: Bioactive components of four Chinese herbs reported in the literature; Table S2: Composition of fatty acids in herb extracts of CHC (CHC-Herb).

## References

[1] Mezes M., Balogh K., Toth K. (2010). Preventive and therapeutic methods against the toxic effects of mycotoxins —A review. Acta Vet. Hung..

[2] Kovesi B., Cserhati M., Erdelyi M., Zandoki E., Mezes M., Balogh K. (2019). Long-Term Effects of Ochratoxin A on the Glutathione Redox System and Its Regulation in Chicken. Antioxidants.

[3] Murugesan G.R., Ledoux D.R., Naehrer K., Berthiller F., Applegate T.J., Grenier B., Phillips T.D., Schatzmayr G. (2015). Prevalence and effects of mycotoxins on poultry health and performance, and recent development in mycotoxin counteracting strategies. Poult. Sci..

[4] Cinar M., Yildirim E., Yigit A.A., Yalcinkaya I., Duru O., Kisa U., Atmaca N. (2014). Effects of dietary supplementation with vitamin C and vitamin E and their combination on growth performance, some biochemical parameters, and oxidative stress induced by copper toxicity in broilers. Biol. Trace Elem. Res..

[5] Pleadin J., Frece J., Markov K. (2019). Mycotoxins in food and feed. Adv. Food Nutr. Res..

[6] Santos R.R., van Eerden E. (2021). Impaired Performance of Broiler Chickens Fed Diets Naturally Contaminated with Moderate Levels of Deoxynivalenol. Toxins.

[7] Burchacka E., Lukaszewicz M., Kulazynski M. (2019). Determination of mechanisms of action of active carbons as a feed additive. Bioorg. Chem..

[8] Bhatti S.A., Khan M.Z., Saleemi M.K., Hassan Z.U. (2021). Combating immunotoxicity of aflatoxin B1 by dietary carbon supplementation in broiler chickens. Environ. Sci. Pollut. Res. Int..

[9] Khatoon A., Khan M.Z., Abidin Z.U., Bhatti S.A. (2018). Effects of feeding bentonite clay upon ochratoxin A-induced immunosuppression in broiler chicks. Food Addit. Contam. Part A.

[10] Oso A.O., Akapo O., Sanwo K.A., Bamgbose A.M. (2014). Utilization of unpeeled cassava (*Manihot esculenta* Crantz) root meal supplemented with or without charcoal by broiler chickens. J. Anim. Physiol. Anim. Nutr..

[11] Schmidt H.P., Hagemann N., Draper K., Kammann C. (2019). The use of biochar in animal feeding. PeerJ.

[12] Kutlu H.R., Unsal I. (1998). Effects of dietary wood charcoal on performance and fatness of broiler chicks. Br. Poult. Sci..

[13] Majewska T., Pyrek D., Faruga A. (2002). A note on the effect of charcoal supplementation on the performance of Big 6 heavy torn turkeys. J. Anim. Feed Sci..

[14] Kihal A., Rodriguez-Prado M., Godoy C., Cristofol C., Calsamiglia S. (2020). In vitro assessment of the capacity of certain mycotoxin binders to adsorb some amino acids and water-soluble vitamins. J. Dairy Sci..

[15] Yildizli G., Coral G., Ayaz F. (2021). Biochar as a Biocompatible Mild Anti-Inflammatory Supplement for Animal Feed and Agricultural Fields. Chem. Biodivers..

[16] Parham S., Kharazi A.Z., Bakhsheshi-Rad H.R., Nur H., Ismail A.F., Sharif S., RamaKrishna S., Berto F. (2020). Antioxidant, Antimicrobial and Antiviral Properties of Herbal Materials. Antioxidants.

[17] Ministry of Agriculture and Rural Affairs of China Announcement No. 194 of the Ministry of Agriculture and Rural Affairs of the People’s Republic of China. http://www.moa.gov.cn/gk/tzgg_1/gg/201907/t20190710_6320678.htm.

[18] Wang L., Gong L., Zhu L., Peng C., Liao J., Ke L., Dong B. (2019). Effects of activated charcoal-herb extractum complex on the growth performance, immunological indices, intestinal morphology and microflora in weaning piglets. RSC Adv..

[19] Yao D., Vlessidis A.G., Gou Y., Zhou X., Zhou Y., Evmiridis N.P. (2004). Chemiluminescence detection of superoxide anion release and superoxide dismutase activity: Modulation effect of *Pulsatilla chinensis*. Anal. Bioanal. Chem..

[20] Ye W., Zhang Q., Hsiao W.W., Zhao S., Che C.T. (2002). New lupane glycosides from *Pulsatilla chinensis*. Planta Med..

[21] Ma Y., Bao Y., Zhang W., Ying X., Stien D. (2020). Four lignans from *Portulaca oleracea* L. and its antioxidant activities. Nat. Prod. Res..

[22] Li C.Y., Meng Y.H., Ying Z.M., Xu N., Hao D., Gao M.Z., Zhang W.J., Xu L., Gao Y.C., Ying X.X. (2016). Three Novel Alkaloids from *Portulaca oleracea* L. and Their Anti-inflammatory Effects. J. Agric. Food Chem..

[23] Wang S., Li J., Sun J., Zeng K.W., Cui J.R., Jiang Y., Tu P.F. (2013). NO inhibitory guaianolide-derived terpenoids from *Artemisia argyi*. Fitoterapia.

[24] Yu C., Chen J., Huang L. (2013). A study on the antitumour effect of total flavonoids from *Pteris multifida* Poir in H22 tumour-bearing mice. Afr. J. Tradit. Complement. Altern. Med..

[25] Liu J., Shu J., Zhang R., Zhang W. (2011). Two new pterosin dimers from *Pteris mutifida* Poir. Fitoterapia.

[26] Wang L., Zhang Y., Guo X., Gong L., Dong B. (2022). Beneficial Alteration in Growth Performance, Immune Status, and Intestinal Microbiota by Supplementation of Activated Charcoal-Herb Extractum Complex in Broilers. Front. Microbiol..

[27] Bakr B.E.A. (2007). The Effect of Using Citrus Wood Charcoal in Broiler Rations on the Performance of Broilers. An-Najah Univ. J. Res..

[28] Kana J., Teguia A., Mungfu B., Tchoumboue J. (2010). Growth performance and carcass characteristics of broiler chickens fed diets supplemented with graded levels of charcoal from maize cob or seed of *Canarium schweinfurthii* Engl. Trop. Anim. Health Prod..

[29] Kutlu H.R., Ünsal I., Görgülü M. (2001). Effects of providing dietary wood (oak) charcoal to broiler chicks and laying hens. Anim. Feed Sci. Technol..

[30] Majewska T., Mikulski D., Siwik T. (2009). Silica grit, charcoal and hardwood ash in turkey nutrition. J. Elem..

[31] Choi J.-S., Jung D.-S., Lee J.-H., Choi Y.-I., Lee J.-J. (2012). Growth performance, immune response and carcass characteristics of finishing pigs by feeding stevia and charcoal. Food Sci. Anim. Resour..

[32] Chu G.M., Kim J.H., Kim H.Y., Ha J.H., Jung M.S., Song Y., Cho J.H., Lee S.J., Ibrahim R.I.H., Lee S.S. (2013). Effects of bamboo charcoal on the growth performance, blood characteristics and noxious gas emission in fattening pigs. J. Appl. Anim. Res..

[33] Kupper T., Fischlin I., Häni C., Spring P. Use of a feed additive based on biochar for mitigation of ammonia emissions from weaned piglets and broilers. Proceedings of the RAMIRAN, 2015—16th International Conference Rural-Urban Symbiosis.

[34] Mekbungwan A., Yamauchi K., Sakaida T. (2004). Intestinal villus histological alterations in piglets fed dietary charcoal powder including wood vinegar compound liquid. Anat. Histol. Embryol..

[35] Sivilai B., Preston T., Leng R., Hang D.T., Linh N.Q. (2018). Rice distillers’ byproduct and biochar as additives to a forage-based diet for growing Moo Lath pigs; effects on growth and feed conversion. Livest. Res. Rural Dev..

[36] Cao J., Li M., Chen J., Liu P., Li Z. (2016). Effects of MeJA on *Arabidopsis* metabolome under endogenous JA deficiency. Sci. Rep..

[37] Taga M.S., Miller E.E., Pratt D.E. (1984). Chia seeds as a source of natural lipid antioxidants. J. Am. Oil Chem. Soc..

[38] Jia Z., Tang M., Wu J. (1999). The determination of flavonoid contents in mulberry and their scavenging effects on superoxide radicals. Food Chem..

[39] Avantaggiato G., Greco D., Damascelli A., Solfrizzo M., Visconti A. (2014). Assessment of multi-mycotoxin adsorption efficacy of grape pomace. J. Agric. Food Chem..

[40] Guo W.R., Ou S.X., Long W.P., Wei Z., Yan X., Yu L. (2015). Simultaneous detection method for mycotoxins and their metabolites in animal urine by using impurity adsorption purification followed by liquid chromatography-tandem mass detection. J. Chromatogr. Sep. Tech..

[41] Shimada K., Fujikawa K., Yahara K., Nakamura T. (1992). Antioxidative Properties of Xanthone on the Auto Oxidation of Soybean in Cylcodextrin Emulsion. J. Agric. Food Chem..

[42] Re R., Pellegrini N., Proteggente A., Pannala A., Yang M., Rice-Evans C. (1999). Antioxidant activity applying an improved ABTS radical cation decolorization assay. Free Radic. Biol. Med..

[43] Jing T., Zhao X. (1995). The improved pyrogallol method by using terminating agent for superoxide dismutase measurement. Prog. Biochem. Biophys..

[44] Seglen P. (1973). Preparation of rat liver cells: III. Enzymatic requirements for tissue dispersion. Exp. Cell Res..

[45] Oetjen E., Schweickhardt C., Unthan-Fechner K., Probst I. (1990). Stimulation of glucose production from glycogen by glucagon, noradrenaline and non-degradable adenosine analogues is counteracted by adenosine and ATP in cultured rat hepatocytes. Biochem. J..

[46] Council N.R. (1994). Nutrient Requirements of Poultry.

[47] Schilling M.W., Radhakrishnan V., Thaxton Y.V., Christensen K., Thaxton J.P., Jackson V. (2008). The effects of broiler catching method on breast meat quality. Meat Sci..

[48] Li K., Zhang Y., Mao Y., Cornforth D., Dong P., Wang R., Zhu H., Luo X. (2012). Effect of very fast chilling and aging time on ultra-structure and meat quality characteristics of Chinese Yellow cattle M. Longissimus lumborum. Meat Sci..

[49] Wardlaw F., McCaskill L., Acton J. (1973). Effect of postmortem muscle changes on poultry meat loaf properties. J. Food Sci..

[50] Cunniff P. (1995). Official Methods of Analysis.

[51] Zheng X., Chen T., Jiang R., Zhao A., Wu Q., Kuang J., Sun D., Ren Z., Li M., Zhao M. (2021). Hyocholic acid species improve glucose homeostasis through a distinct TGR5 and FXR signaling mechanism. Cell Metab..

[52] Fricke R.F., Jorge J. (1990). Assessment of efficacy of activated charcoal for treatment of acute T-2 toxin poisoning. J. Toxicol. Clin. Toxicol..

[53] Fink-Gremmels J., Georgiou N. (1996). Risk assessment of mycotoxins for the consumer. Residues of Veterinary Drugs and Mycotoxins in Animal Products.

[54] Reverberi M., Ricelli A., Zjalic S., Fabbri A.A., Fanelli C. (2010). Natural functions of mycotoxins and control of their biosynthesis in fungi. Appl. Microbiol. Biotechnol..

[55] (2018). Hygienical Standard for Feeds. General Administration of Quality Supervision, Inspection and Quarantine of the People’s Republic of China: Beijing, China.

[56] Surai P.F., Mezes M., Melnichuk S.D., Fotina T.I. (2008). Mycotoxins and animal health: From oxidative stress to gene expression. Krmiva Časopis Hranidbi Zivotinja Proizv. Tehnol. Krme.

[57] Peraica M., Radic B., Lucic A., Pavlovic M. (1999). Toxic effects of mycotoxins in humans. Bull. World Health Organ..

[58] Akbarian A., Michiels J., Degroote J., Majdeddin M., Golian A., De Smet S. (2016). Association between heat stress and oxidative stress in poultry; mitochondrial dysfunction and dietary interventions with phytochemicals. J. Anim. Sci. Biotechnol..

[59] Adams C.A. (2006). Nutrition-based health in animal production. Nutr. Res. Rev..

[60] Vinus R.D., Sheoran N., Maan N., Tewatia B. (2018). Potential benefits of herbal supplements in poultry feed: A review. Pharma Innov..

[61] Dan Z. (2006). Study on antimicrobial effect of flavonoids from *Portulace oleracea* L.. J. Anhui Agric. Sci..

[62] Gonnella M., Charfeddine M., Conversa G., Santamaria P. (2010). Purslane: A review of its potential for health and agricultural aspects. Eur. J. Plant Sci. Biotechnol..

[63] Harinantenaina L., Matsunami K., Otsuka H. (2008). Chemical and biologically active constituents of *Pteris multifida*. J. Nat. Med..

[64] Iranshahy M., Javadi B., Iranshahi M., Jahanbakhsh S.P., Mahyari S., Hassani F.V., Karimi G. (2017). A review of traditional uses, phytochemistry and pharmacology of *Portulaca oleracea* L.. J. Ethnopharmacol..

[65] Jin H.Z., Lee J.H., Lee D., Hong Y.S., Kim Y.H., Lee J.J. (2004). Inhibitors of the LPS-induced NF-kappaB activation from *Artemisia Sylvatica*. Phytochemistry.

[66] Liu T., Ye L., Guan X., Liang X., Li C., Sun Q., Liu Y., Chen S., Bang F., Liu B. (2013). Immunopontentiating and antitumor activities of a polysaccharide from *Pulsatilla chinensis* (Bunge) Regel. Int. J. Biol. Macromol..

[67] Mimaki Y., Kuroda M., Asano T., Sashida Y. (1999). Triterpene saponins and lignans from the roots of *Pulsatilla chinensis* and their cytotoxic activity against HL-60 cells. J. Nat. Prod..

[68] Seo J.-M., Kang H.-M., Son K.-H., Kim J.H., Lee C.W., Kim H.M., Chang S.-I., Kwon B.-M. (2003). Antitumor activity of flavones isolated from *Artemisia argyi*. Planta Med..

[69] Sun Y., Liu J., Yu H., Gong C. (2010). Isolation and evaluation of immunological adjuvant activities of saponins from the roots of *Pulsatilla chinensis* with less adverse reactions. Int. Immunopharmacol..

[70] Wen J., Shi H., Xu Z., Chang H., Jia C., Zan K., Jiang Y., Tu P.F. (2010). Dimeric guaianolides and sesquiterpenoids from *Artemisia anomala*. J. Nat. Prod..

[71] Zhou F., Lv O., Zheng Y., Wang J., Hu P., Wang Z., Yang L. (2012). Inhibitory effect of *Pulsatilla chinensis* polysaccharides on glioma. Int. J. Biol. Macromol..

[72] Zhou Y.X., Xin H.L., Rahman K., Wang S.J., Peng C., Zhang H. (2015). *Portulaca oleracea* L.: A review of phytochemistry and pharmacological effects. BioMed Res. Int..

[73] Zhang J., Pavek P., Kamaraj R., Ren L., Zhang T. (2021). Dietary phytochemicals as modulators of human pregnane X receptor. Crit. Rev. Food Sci. Nutr..

[74] Zhang Z.Y., Wang Y.G., Huang X.Y., Wang M.X., Yang L., Ma Z.C., Tang X.L., Gao Y. (2018). Effect of six components in Polygoni Multiflori Radix on regulation of CYP3A4 mediated by human pregnane X receptor. Zhongguo Zhong Yao Za Zhi.

[75] Liu Y.H., Mo S.L., Bi H.C., Hu B.F., Li C.G., Wang Y.T., Huang L., Huang M., Duan W., Liu J.P. (2011). Regulation of human pregnane X receptor and its target gene cytochrome P450 3A4 by Chinese herbal compounds and a molecular docking study. Xenobiotica.

[76] Modarai M., Suter A., Kortenkamp A., Heinrich M. (2011). The interaction potential of herbal medicinal products: A luminescence-based screening platform assessing effects on cytochrome P450 and its use with devil’s claw (*Harpagophyti radix*) preparations. J. Pharm. Pharmacol..

[77] Kliewer S.A. (2015). Nuclear receptor PXR: Discovery of a pharmaceutical anti-target. J. Clin. Investig..

[78] Chen Y., Tang Y., Guo C., Wang J., Boral D., Nie D. (2012). Nuclear receptors in the multidrug resistance through the regulation of drug-metabolizing enzymes and drug transporters. Biochem. Pharmacol..

[79] Krezel W., Ruhl R., de Lera A.R. (2019). Alternative retinoid X receptor (RXR) ligands. Mol. Cell. Endocrinol..

[80] Modica S., Bellafante E., Moschetta A. (2009). Master regulation of bile acid and xenobiotic metabolism via the FXR, PXR and CAR trio. Front. Biosci. (Landmark Ed.).

[81] Bligh E.G., Dyer W.J. (1959). A rapid method of total lipid extraction and purification. Can. J. Biochem. Physiol..

[82] Ma C., Liu Y., Liu S., Levesque C.L., Zhao F., Yin J., Dong B. (2020). Branched chain amino acids alter fatty acid profile in colostrum of sows fed a high fat diet. J. Anim. Sci. Biotechnol..

[83] Qu W.X., Mou Z.L., Cui H.Y., Zhang Z.Q. (2011). Analysis of fatty acids in *A. szechenyianum* Gay. by microwave-assisted extraction and gas chromatography-mass spectrometry. Phytochem. Anal..

[84] Neuvonen P.J., Kuusisto P., Manninen V., Vapaatalo H., Miettinen T.A. (1989). The mechanism of the hypocholesterolaemic effect of activated charcoal. Eur. J. Clin. Investig..

[85] Zhang M., Xie Z., Gao W., Pu L., Wei J., Guo C. (2016). Quercetin regulates hepatic cholesterol metabolism by promoting cholesterol-to-bile acid conversion and cholesterol efflux in rats. Nutr. Res..

[86] Hoang M.H., Jia Y., Mok B., Jun H.J., Hwang K.Y., Lee S.J. (2015). Kaempferol ameliorates symptoms of metabolic syndrome by regulating activities of liver X receptor-beta. J. Nutr. Biochem..

[87] Chavez-Santoscoy R.A., Gutierrez-Uribe J.A., Granados O., Torre-Villalvazo I., Serna-Saldivar S.O., Torres N., Palacios-Gonzalez B., Tovar A.R. (2014). Flavonoids and saponins extracted from black bean (*Phaseolus vulgaris* L.) seed coats modulate lipid metabolism and biliary cholesterol secretion in C57BL/6 mice. Br. J. Nutr..

[88] Tan Z., Luo M., Yang J., Cheng Y., Huang J., Lu C., Song D., Ye M., Dai M., Gonzalez F.J. (2016). Chlorogenic acid inhibits cholestatic liver injury induced by alpha-naphthylisothiocyanate: Involvement of STAT3 and NFkappaB signalling regulation. J. Pharm. Pharmacol..

[89] Yang T.T., Koo M.W. (2000). Chinese green tea lowers cholesterol level through an increase in fecal lipid excretion. Life Sci..

[90] Blumberg B., Sabbagh W., Juguilon H., Bolado J., van Meter C.M., Ong E.S., Evans R.M. (1998). SXR, a novel steroid and xenobiotic-sensing nuclear receptor. Genes Dev..

[91] Kliewer S.A., Moore J.T., Wade L., Staudinger J.L., Watson M.A., Jones S.A., McKee D.D., Oliver B.B., Willson T.M., Zetterstrom R.H. (1998). An orphan nuclear receptor activated by pregnanes defines a novel steroid signaling pathway. Cell.

[92] Delfosse V., Huet T., Harrus D., Granell M., Bourguet M., Gardia-Parege C., Chiavarina B., Grimaldi M., Le Mevel S., Blanc P. (2021). Mechanistic insights into the synergistic activation of the RXR-PXR heterodimer by endocrine disruptor mixtures. Proc. Natl. Acad. Sci. USA.

[93] Modica S., Gadaleta R.M., Moschetta A. (2010). Deciphering the nuclear bile acid receptor FXR paradigm. Nucl. Recept. Signal..

[94] Jung D., Mangelsdorf D.J., Meyer U.A. (2006). Pregnane X receptor is a target of farnesoid X receptor. J. Biol. Chem..

[95] Kakizaki S., Yamazaki Y., Takizawa D., Negishi M. (2008). New insights on the xenobiotic-sensing nuclear receptors in liver diseases--CAR and PXR. Curr. Drug Metab..

[96] Wu Q., Dohnal V., Huang L., Kuca K., Yuan Z. (2010). Metabolic pathways of trichothecenes. Drug Metab. Rev..

[97] Handschin C., Meyer U.A. (2003). Induction of drug metabolism: The role of nuclear receptors. Pharmacol. Rev..

[98] D’Archivio M., Filesi C., Vari R., Scazzocchio B., Masella R. (2010). Bioavailability of the polyphenols: Status and controversies. Int. J. Mol. Sci..

[99] Yu Z., Yue B., Ding L., Luo X., Ren Y., Zhang J., Mani S., Wang Z., Dou W. (2020). Activation of PXR by Alpinetin Contributes to Abrogate Chemically Induced Inflammatory Bowel Disease. Front. Pharmacol..

[100] Shao Y.Y., Guo Y., Feng X.J., Liu J.J., Chang Z.P., Deng G.F., Xu D., Gao J.P., Hou R.G. (2021). Oridonin Attenuates TNBS-induced Post-inflammatory Irritable Bowel Syndrome via PXR/NF-kappaB Signaling. Inflammation.

[101] Tafazoli S., Mashregi M., O’Brien P.J. (2008). Role of hydrazine in isoniazid-induced hepatotoxicity in a hepatocyte inflammation model. Toxicol. Appl. Pharmacol..

[102] Winterbourn C.C., Hampton M.B., Livesey J.H., Kettle A.J. (2006). Modeling the reactions of superoxide and myeloperoxidase in the neutrophil phagosome: Implications for microbial killing. J. Biol. Chem..

[103] Soglia F., Petracci M., Davoli R., Zappaterra M. (2021). A critical review of the mechanisms involved in the occurrence of growth-related abnormalities affecting broiler chicken breast muscles. Poult. Sci..

[104] Purslow P.P., Gagaoua M., Warner R.D. (2021). Insights on meat quality from combining traditional studies and proteomics. Meat Sci..

[105] Yamauchi K., Manabe N., Matsumoto Y., Takenoyama S., Yamauchi K.E. (2014). Increased collagen III in culled chicken meat after feeding dietary wood charcoal and vinegar contributes to palatability and tenderness. Anim. Sci. J..

[106] Muzolf-Panek M., Kaczmarek A. (2021). Chemometric Analysis of Fatty Acid Composition of Raw Chicken, Beef, and Pork Meat with Plant Extract Addition during Refrigerated Storage. Molecules.

[107] Arshad M.S., Anjum F.M., Asghar A., Khan M.I., Yasin M., Shahid M., El-Ghorab A.H. (2011). Lipid stability and antioxidant profile of microsomal fraction of broiler meat enriched with alpha-lipoic acid and alpha-tocopherol acetate. J. Agric. Food Chem..

[108] Mancini R., Hunt M. (2005). Current research in meat color. Meat Sci..

[109] Suman S.P., Joseph P. (2013). Myoglobin chemistry and meat color. Annu. Rev. Food Sci. Technol..

[110] Hughes J.M., Clarke F.M., Purslow P.P., Warner R.D. (2020). Meat color is determined not only by chromatic heme pigments but also by the physical structure and achromatic light scattering properties of the muscle. Compr. Rev. Food Sci. Food Saf..

