# Supplementary material for: Cooperative Interaction of Phenolic Acids and Flavonoids Contained in Activated Charcoal with Herb Extracts, Involving Cholesterol, Bile Acid, and FXR/PXR Activation in Broilers Fed with Mycotoxin-Containing Diets
Source: Antioxidants (Basel). 2022 Nov 7;11(11):2200. doi: 10.3390/antiox11112200 (PMC9686537; doi:10.3390/antiox11112200)
Supplement: Supplementary file 1 [file antioxidants-11-02200-s001.zip › antioxidants-1988986-supplementary.pdf]

antioxidants

MDPI

Table S1. Bioactive components of four Chinese herbs reported in literatures.

Name	Components	Functions	References
Pulsatilla chinensis	Purified polysaccharides	Antitumor	[1,2]
	Triterpene saponins	Antibacteria; anti-inflammatory	[3-5]
	Lignan, (+)-pinoresinol, β-peltatin	Cytoxic to leukemia cells	[3]
	Water extracts	Increase SOD activity in liver	[6]
Portulaca oleracea L.	Flavonoids	Anti-microbes	[7]
	Phenolics, α-tocopherol, β-carotene, ascorbic acid, and glutathione	Antioxidants	[8,9]
	Polysaccharides	Anti-inflammatory	[10]
Artemisia argyi Folium	Ethanol extracts: Guaianolide sesquiterpene lactones, sesquiterpenes, artemisolide, 3-methoxytanapartholide, deacetyllaurenobiolide, moxartenolide, arteminolides	Anti-inflammatory	[11,12]
	Flavones	Anti-tumor	[13]
Pteris multifida Poir	1-butanol-soluble fraction	Cytoxic to tumor KB cells	[14]
	Ethanol extracts: Sesquiterpenoids and ent-kaurane diterpenoids	Cytoxic to tumor cells	
	Flavonoids	Anti-tumor	[15]

Table S2. Composition of fatty acids in herb extracts of CHC (CHC-Herb)[1,2].

Fatty acids	Formula	Molecular Weight	Percentage Content (%)
Capric acid	C10:0	172	1.48
Lauric acid	C12:0	200	2.9
4-dodecenoic acid	C12:1	198	1.69
Myristic acid	C14:0	228	1.29
5-tetradecenoic acid	C14:1	226	0.96
Palmitic acid	C16:0	256	5.37
7-hexadeenoic acid	C16:1	254	1.63
7,10-hexadecadienoic acid	C16:2	252	5.89
7,10,13-hexadecatrienoic acid	C16:3	250	3.38
Stearic acid	C18:0	284	1.76
9-octadecenoic acid	C18:1	282	1.74
9,12-octadecadienoic acid	C18:2	280	7.51
Alpha-Linolenic acid	C18:3	278	7.65
5,9,12-octadecatrienoic acid	C18:3	278	1.6
Arachidic acid	C20:0	312	2.3
11,14-eicosadienoic acid (Eicosadienoic acid)	C20:2	308	14.85
Arachidonic Acid	C20:4	304	16.21
Behenic acid	C22:0	340	3.52

13-docosenoic acid	C22:1	338	6.49
Lignoceric acid	C24:0	368	1.58
Saturated fatty acids			20.2
Monosaturated fatty acids			12.51
Polyunsaturated fatty acids			57.09

[1]Note: Total lipids were extracted and quantified following the method reported by Bligh [16]. Fatty acid (FA) analysis was carried out using the reported method of gas chromatography with modification [17,18]. Briefly, the herb extracts (CHC-Herb) were dissolved in 10 mL of methanol. Then 1 mL of internal standard solution and 10 mL of boron trifluoride–methanol were added to 1 mL of the extract solution, and the mixture was boiled in a water bath for 10 min. The derivatization reaction was stopped by adding 2 mL of saturated sodium chloride solution. After cooling, the fatty acid methyl esters (FAME) were extracted with 10 mL of hexane and 1 mL of the hexane extraction was quantified using a Shidmadzu 2010 gas chromatograph equipped with a 50-mm capillary column (0.32-mm internal diameter) coated with BPX-70 (0.25-µm film thickness; SGE Pty Ltd., Ringwood, VIC, Australia). Each sample (1 µL) was then injected in to the column using an automatic injector (Shimadzu AOC 20i, Shimadzu Corporation, Kyoto, Japan) at a split ratio of 20:1. Identification of FA peaks was made by comparing their retention times to that of known FAME standards (Sigma-Aldrich, St Louis, MO, USA). C11:0 was set as the internal control. Individual FA peaks were quantified as percentage of the total area.
[2]Total fat content of CHC-Herb was 10 mg per gram extract (10 mL CHC-Herb). Total fatty acids accounted for 15% of total fat. Polyunsaturated fatty acids (PUFA) were taken up 57.09% of total fatty acids. Thus, in CHC-Herb, PUFA content accounts for 45 µg per gram extract (per mL of CHC-Herb).

References

1. Liu, T.; Ye, L.; Guan, X.; Liang, X.; Li, C.; Sun, Q.; Liu, Y.; Chen, S.; Bang, F.; Liu, B. Immunopontentiating and antitumor activities of a polysaccharide from Pulsatilla chinensis (Bunge) Regel. *International Journal of Biological Macromolecules* **2013**, *54*, 225-229.
2. Zhou, F.; Lv, O.; Zheng, Y.; Wang, J.; Hu, P.; Wang, Z.; Yang, L. Inhibitory effect of Pulsatilla chinensis polysaccharides on glioma. *International Journal of Biological Macromolecules* **2012**, *50*, 1322-1326.
3. Mimaki, Y.; Kuroda, M.; Asano, T.; Sashida, Y. Triterpene saponins and lignans from the roots of Pulsatilla chinensis and their cytotoxic activity against HL-60 cells. *Journal of natural products* **1999**, *62*, 1279-1283.
4. Sun, Y.; Liu, J.; Yu, H.; Gong, C. Isolation and evaluation of immunological adjuvant activities of saponins from the roots of Pulsatilla chinensis with less adverse reactions. *International Immunopharmacology* **2010**, *10*, 584-590.
5. Ye, W.; Zhang, Q.; Hsiao, W.W.; Zhao, S.; Che, C.-T. New lupane glycosides from Pulsatilla chinensis. *Planta medica* **2002**, *68*, 183-186.
6. Yao, D.; Vlessidis, A.G.; Gou, Y.; Zhou, X.; Zhou, Y.; Evmiridis, N.P. Chemiluminescence detection of superoxide anion release and superoxide dismutase activity: modulation effect of Pulsatilla chinensis. *Analytical and bioanalytical chemistry* **2004**, *379*, 171-177.
7. Dan, Z. Study on antimicrobial effect of flavonoids from Portulace oleracea L. *Journal of Anhui Agricultural Sciences* **2006**, *34*, 7.
8. Gonnella, M.; Charfeddine, M.; Conversa, G.; Santamaria, P. Purslane: a review of its potential for health and agricultural aspects. *The European Journal of Plant Science and Biotechnology* **2010**, *4*, 131-136.
9. Iranshahy, M.; Javadi, B.; Iranshahi, M.; Jahanbakhsh, S.P.; Mahyari, S.; Hassani, F.V.; Karimi, G. A review of traditional uses, phytochemistry and pharmacology of Portulaca oleracea L. *Journal of ethnopharmacology* **2017**, *205*, 158-172.
10. Zhou, Y.-X.; Xin, H.-L.; Rahman, K.; Wang, S.-J.; Peng, C.; Zhang, H. Portulaca oleracea L.: a review of phytochemistry and pharmacological effects. *BioMed research international* **2015**, *2015*.
11. Jin, H.Z.; Lee, J.H.; Lee, D.; Hong, Y.S.; Kim, Y.H.; Lee, J.J. Inhibitors of the LPS-induced NF-κB activation from Artemisia sylvatica. *Phytochemistry* **2004**, *65*, 2247-2253.

12. Wen, J.; Shi, H.; Xu, Z.; Chang, H.; Jia, C.; Zan, K.; Jiang, Y.; Tu, P. Dimeric guaianolides and sesquiterpenoids from Artemisia anomala. *Journal of natural products* **2010**, *73*, 67-70.
13. Seo, J.-M.; Kang, H.-M.; Son, K.-H.; Kim, J.H.; Lee, C.W.; Kim, H.M.; Chang, S.-I.; Kwon, B.-M. Antitumor activity of flavones isolated from Artemisia argyi. *Planta medica* **2003**, *69*, 218-222.
14. Harinantenaina, L.; Matsunami, K.; Otsuka, H. Chemical and biologically active constituents of Pteris multifida. *Journal of natural medicines* **2008**, *62*, 452-455.
15. Yu, C.; Chen, J.; Huang, L. A study on the antitumour effect of total flavonoids from Pteris multifida Poir in H22 tumour-bearing mice. *African Journal of Traditional, Complementary and Alternative Medicines* **2013**, *10*, 459-463.
16. Bligh, E.G., Dyer, W.J. A rapid method of total lipid extraction and purification. *Canadian Journal of Biochemistry and Physiology* **1959**, 37, 911-917.
17. Qu, W.X.; Mou, Z.L.; Cui, H.Y.; Zhang, Z.Q. Analysis of fatty acids in A. szechenyianum Gay. by microwave-assisted extraction and gas chromatography-mass spectrometry. *Phytochemical analysis : PCA* **2011**, *22*, 199-204.
18. Ma, C.; Liu, Y.; Liu, S.; Levesque, C.L.; Zhao, F.; Yin, J.; Dong, B. Branched chain amino acids alter fatty acid profile in colostrum of sows fed a high fat diet. *Journal of Animal Science and Biotechnol* **2020**, *11*, 9.
